# Developmentally timed sensory integration enables efficient larval dispersal

**DOI:** 10.1016/j.isci.2026.117222

**Published:** 2026-08-13

**Authors:** Marte Lønnum, Madeline M. Schuldt, Johan Hovland, Jose Davila-Velderrain, Lena van Giesen

**Affiliations:** 1 Department of Biology, Norwegian University of Science and Technology, 7491 Trondheim, Norway; 2 Human Technopole, Viale Rita Levi-Montalcini 1, 20157 Milan, Italy

**Keywords:** sensory biology, planula larvae, locomotion, cilia

## Abstract

Animals integrate internal and external sensory information to adjust movement according to the physical constraints imposed by body-environment interactions. Efficient locomotion is especially important in small organisms where the motile phase is ontogenetically restricted. Motile larvae of otherwise sessile cnidarians must disperse and identify a suitable habitat in a restricted time frame. Here we show that dispersal in *Nematostella* larvae is accomplished by a constant ciliary sensory-motor system that produces stimulus-induced movement. In contrast, neuromuscular and sensory systems gradually increase in complexity during development, enabling movement-associated gait control through matching reafferent (self-generated) and external sensory information. Together with ciliary propulsion, the developmentally timed appearance of sensory and neuronal structures endows the animal with the ability to integrate information to shape swimming behavior and achieve efficient dispersal in a timely manner, appropriate to the physical challenges of its specific ecological niche.

## Highlights

• Swimming and sensory perception in *Nematostella* larvae change during development
• Specialized sensory and motor cilia appear at different developmental time points
• Cilia and nervous system work in concert to optimize swimming behavior
• Efficient dispersal is achieved through enhanced perception and straight swimming

## Graphical abstract

**Figure:**
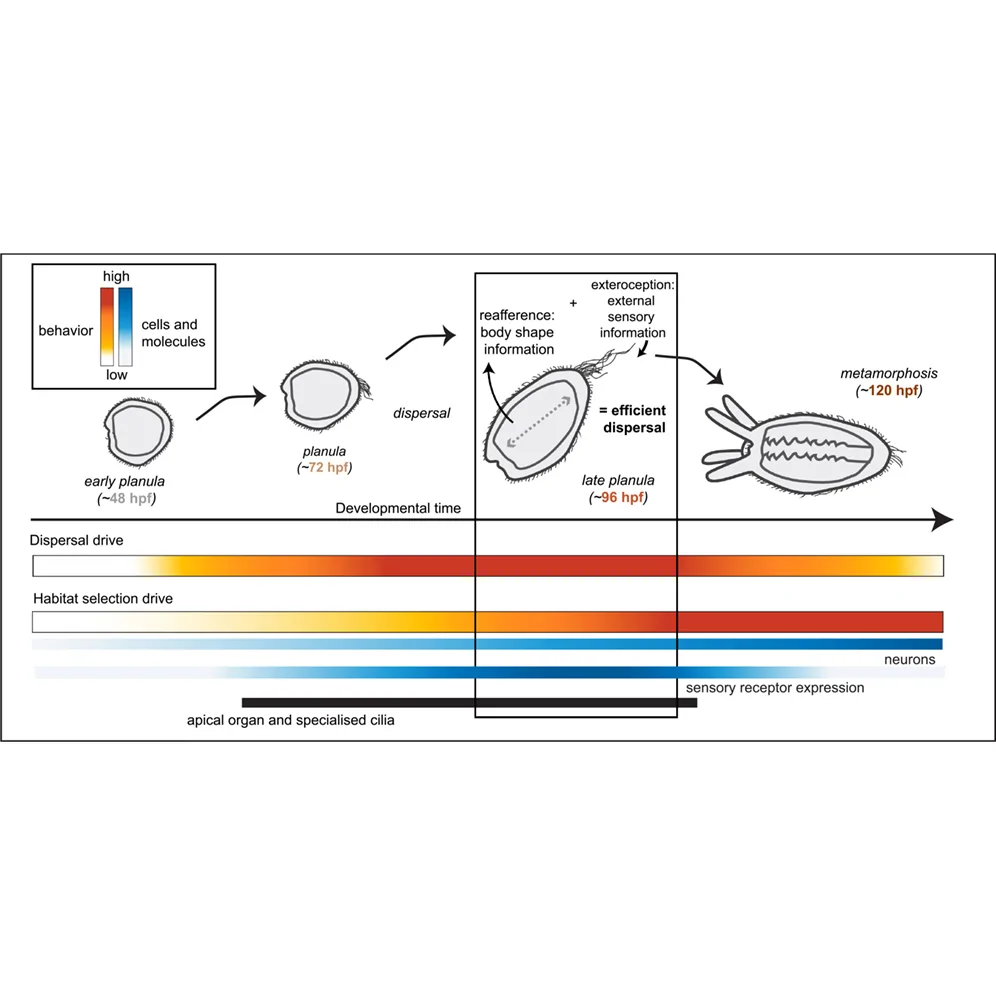

## Teaser

Systems neuroscience; Evolutionary biology; Developmental biology

## Introduction

Anthozoan cnidarians such as anemones and corals are sessile or semi-sessile marine invertebrates. Their complex life cycle includes a motile larval stage which serves dispersal, an important feature that ensures the resilience of the species by enhancing genetic diversity and facilitating inhabitation of novel ecological niches.^1^^,^^2^ During the larval phase, the animals display state-specific behaviors, with planktonic larvae exhibiting dispersal behavior early in development. Animals can disperse and explore the environment passively (e.g., by ocean currents) or actively (through swimming). The second type of behavior occurs when the larvae become competent, a physiological state in which behavior centers around the choice of a permanent settlement site. Many aspects of larval dispersal and settlement remain enigmatic. It is not well understood which sensory modalities cnidarian larvae employ to guide swimming or settlement, how such information is processed, how it modifies behavior, and which cellular subsystems are involved.

Anthozoan larvae are diverse in developmental time and anatomy,^3^ and consequently larval behavior too is multifaceted and species-dependent, suggesting that both these traits contribute to adaptations to a specialized ecological niche with its physical peculiarities. Swimming in the larvae can encompass a variety of modes and planes, and some cnidarian larvae even exhibit other complex behaviors such as active predation.^4^^,^^5^^,^^6^ Within the same species, larval behavior is highly plastic throughout development, with animals transitioning from near motionless to active swimming and later to crawling and gliding or probing the substrate immediately prior to settlement.^7^^,^^8^^,^^9^

For both dispersal and settlement, it can be assumed that sensory information is relevant in guiding the larvae. Indeed, both behaviors have been linked to sensory perception in cnidarian planula larvae.^6^^,^^10^^,^^11^^,^^12^^,^^13^ Cnidarian larvae have not been found to perform active taxis but rather phobic responses which nevertheless result in specific distribution of the adult polyps with respect to sensory stimuli.^13^ During these phobic behaviors, the animals respond to sensory stimuli by modifying ciliary beating and body shape, which result in slow positional changes.^6^^,^^8^^,^^11^ Using phobic responses rather than active tactile swimming is a sensible and energy-efficient strategy, considering that, with the size of the larvae and the vastness of the ocean, active swimming toward or away from a stimulus would often exceed the physiological capacity of the animals.^14^

How cnidarian larvae with their small size and relatively simple body plan regulate these diverse behaviors is unclear. The motile planula larva is polarized and swims forward with its aboral end propelled by cilia, which cover the whole animal, forming metachronal waves that improve the efficiency of locomotion.^15^^,^^16^ In addition, some anthozoan cnidarians possess a sensory organ, the apical organ, with long protruding cilia at the aboral end.^17^^,^^18^^,^^19^ Furthermore, many other specialized cell types, such as secretory, neurosecretory, putative sensory, and support cells are enriched in the apical region of the larvae, suggesting that this area combines several sensory effector centers that may be involved in guiding specific behaviors during the larval life.^19^^,^^20^^,^^21^^,^^22^ This idea is also supported by the underlying prominent aboral nerve plexus, of which some parts might be larval-specific.^17^^,^^20^^,^^23^ In addition to the above-mentioned cell types, muscles become more prevalent and increasingly organized over the course of development,^24^ and late-stage planula larvae can contract using their muscular-hydraulic system.^6^^,^^25^

Knowledge about cnidarian behavior is critical to our understanding of the basic biology of this vast group of marine invertebrates that form the foundation of many rich ecosystems. Cnidarian planula larvae also occupy a particularly interesting evolutionary position, being one of the most basal extant animal lineages with a nervous system that might influence their behavioral repertoire already during early life stages. While even single-celled eukaryotes can perform sophisticated cilia-mediated behaviors^26^^,^^27^ and sponge larvae use both sensory and ciliated cells to modify their swimming pattern in response to light,^28^ it is an exciting endeavor to study the exact function of the nervous system and other larval-specific structures, such as the apical organ, to shed light on the complex interplay between cilia and developing nervous and muscular systems.

**Figure 1:**
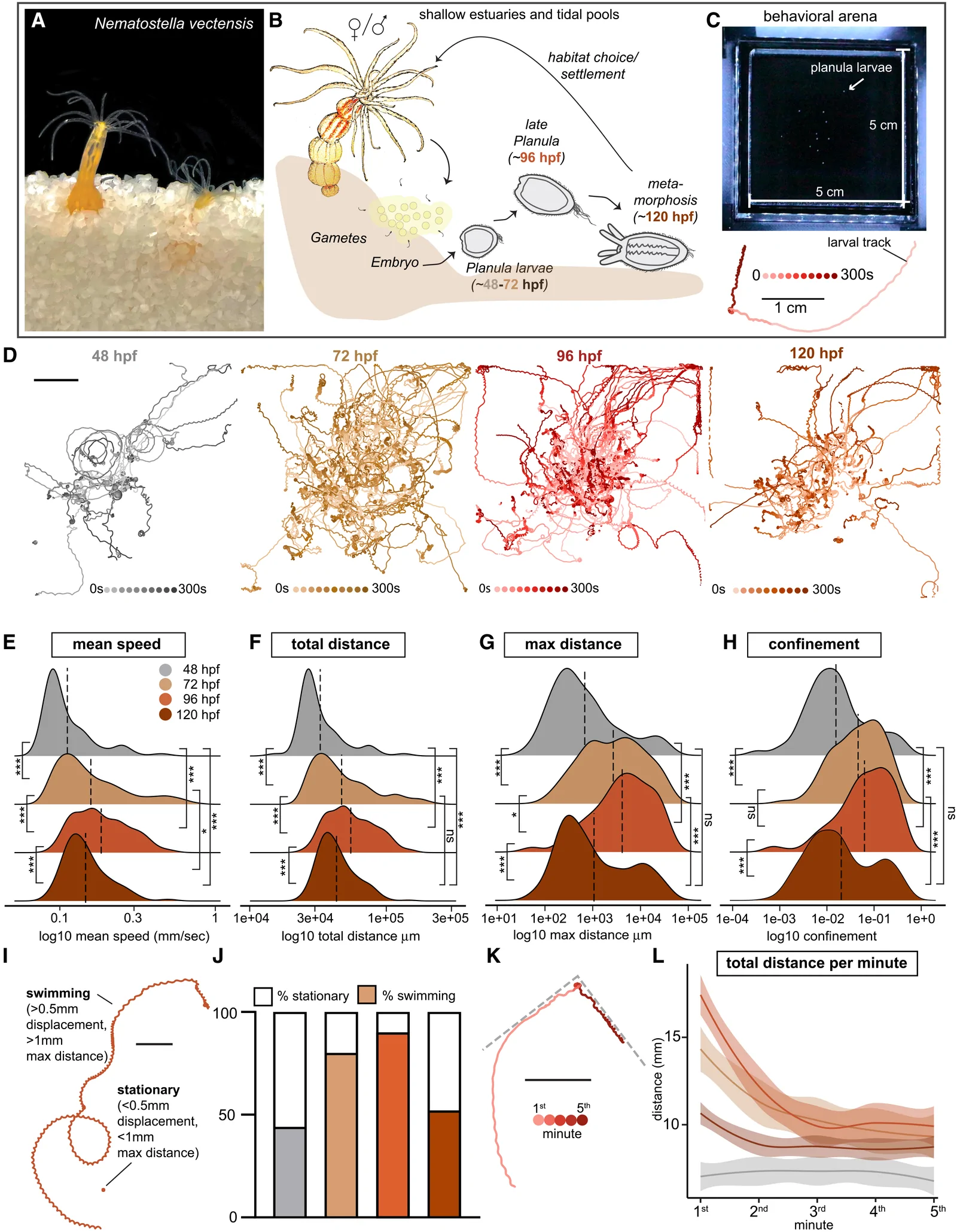
Swimming behavior is changing over the course of larval development (A) *Nematostella vectensis* adult, (B) its life cycle, and (C) the behavioral arena (5 × 5 cm) used in the horizontal swimming experiments and an example of a swimming track obtainable in this assay. (D) Overlaid tracks produced during a 5-min video from four ages of *N. vectensis* larvae showing swimming behavior. 48 hpf (*n* = 123, *N* = 3), 72 hpf (*n* = 167, *N* = 3), 96 hpf (*n* = 158, *N* = 3) and 120 hpf (*n* = 187, *N* = 3), scale bar, 1 cm. (E–H) Distributions of track mean speed, total distance, maximum distance and confinement ratio. Dashed line at the mean; stars denote statistical significance of ns *p* > 0.05, ∗*p* < 0.05, ∗∗*p* < 0.01, and ∗∗∗*p* < 0.001 by Kolmogorov-Smirnov test, x axes on log10 scale. (I) The defined thresholds for categorizing larvae as either stationary or moving, scale bar, 5 mm. (J) Ratio of moving and stationary larvae per age (48 hpf: 44% moving, 72 hpf: 80% moving, 96 hpf: 89% moving, 120 hpf: 52% moving). (K) Example trace (scale bar, 1 cm) and (L) calculations of age-specific average distances the larvae swam per minute. Lines show a smoothed conditional mean line ± SEM. See also Tables S1–S6.

Here we describe the basic swimming behavior of the *Nematostella vectensis* planula larvae over the course of development from hatching to metamorphosis into a primary polyp. To study if swimming behavior indeed changes over development and reflects life stage-specific behavioral interests that are mediated by specialized anatomical features, we analyzed these aspects concomitantly in carefully selected stages. *Nematostella* is a burrowing sea anemone, commonly found in shallow estuaries and tidal pools. Its life cycle includes a motile larval stage with an apical tuft that transitions into a primary polyp (metamorphosis) within a few days (Figures 1A and 1B). Our data show that swimming behavior changes drastically over the course of development. *Nematostella* planula larvae become faster and swim further and are less spatially confined until metamorphosis. This advance in swimming abilities occurs simultaneously with an increase in sensory capabilities, in the form of a higher response to mechanical perturbation, a longer apical tuft, and the enhanced expression of sensory receptors, which were found predominantly in the ciliated epithelial cells. Furthermore, while the length of the motile cilia and the body remain relatively stable during the active phase, the animal’s body shape changes both over development and as the animals behave, correlating with specific swimming modes. We find that the nervous system controls the shape-shifting abilities of the late larval stages but has little impact on sensory perception or the ciliary beating per se, suggesting that *Nematostella* larvae use two independent systems to control swimming behavior: a ciliary sensory-motor system and a neuronally controlled muscular contraction system, aimed at optimizing body shape to swimming mode.

## Results

### Swimming behavior changes over the course of larval development

After preliminary experiments, we chose four ages for behavioral observations at which animals showed clearly distinct behavioral patterns. These timepoints roughly correspond to two phases of the early planula (48 and 72 h postfertilization (hpf)), the late planula stage (96 hpf), and the tentacle bud or metamorphosed stage (120 hpf), at our developmental conditions (21 °C) (Figure 1B). To assess detailed aspects of behavior at these ages, we developed an assay that allowed us to obtain comprehensive behavioral tracks and the associated metrics under standardized conditions by filming as the animals moved freely for 5 min (Figures 1C, S1A, and S1B, see STAR Methods section for details). As previously reported,^8^ larvae quickly develop into active swimmers between 48 and 72 hpf and become more explorative at 96 hpf before mostly ceasing activity around 120 hpf (Figure 1D). Our tracked data allowed us to extract detailed aspects of the swimming behavior as the animals developed. For example, we observed that not all metrics changed simultaneously or stereotypically between and within the different ages, indicating that behavioral control is more complex than previously appreciated and may depend on different aspects of larval development, specifically anatomical characteristics and information processing capacity.

As they age, more larvae swim faster and further, with the average farthest distance (59.5 mm) and highest mean speed (0.2 mm/s) occurring at 96 hpf; however, the individual with the highest total distance (224.5 mm) and mean speed (0.75 mm/s) was a 72 hpf-aged individual. The slowest age was 48 hpf (mean speed 0.12 mm/s), which also swam the shortest average distance (35.8 mm). At 120 hpf these values drop (mean speed 0.15 mm/s and total distance 45.5 mm) and reach similar values to those observed in 72 hpf larvae (Figures 1E–1H, S1C, and S1E; Tables S1–S4). The total distance traveled correlates with speed (*R*^2^: 48 hpf: 0.948, 72 hpf: 0.998, 96 hpf: 0.998, 120 hpf: 0.968), as these metrics depend on each other (Figures 1E and S1J, see also STAR Methods). However, total distance is relative (larvae could have a high total distance value by swimming spatially confined in circles), which would not contribute to an actual Euclidean distance, a value that is more relevant for biological phenomena such as dispersal behavior. To determine if larvae also travel further in space and do so more effectively, we measured maximum distance (Euclidean distance Figure 1G) and confinement as a measure of efficiency to reach a distant point in space (Figures 1H, S1F, and S1I and STAR Methods for calculations). Animals at 48 hpf were most confined on average (ratio 0.048) and displayed the lowest maximum distance (3.1 mm). In contrast, 96 hpf larvae showed lower confinement (ratio 0.12) and the highest average maximum distance (8.7 mm). Interestingly, when looking at the distribution of these two metrics, we found that 48 and 120 hpf old larvae show distributions with mostly low, but subpopulations of relatively high values. This pattern was not found at 72 and 96 hpf, which display a unimodal distribution of similarly high values, indicating that all larvae at these ages and only a small proportion of the youngest and oldest larvae are capable of dispersing efficiently. Intriguingly, these observations parallel the appearance of the apical tuft, a larval-specific sensory structure.^29^

As previously reported,^8^^,^^11^ we also observed that *Nematostella* larvae can switch between stationary and swimming modes (Figure 1I), indicating that active swimming behavior is an energetically costly behavior that the larvae will only display when conditions are advantageous, for example for dispersing away from the colony, or when conditions are so unfavorable that the larvae or the settled polyp risk physical damage (such as high UV intensity^11^^,^^13^). When quantifying the percentage of stationary versus moving individuals across age, we found that the ratio is shifting in favor of movement in 72 (80%) and 96 hpf (89%), while for the youngest and oldest larvae the ratio is approximately 50% between stationary and moving individuals (Figure 1J; Table S5).

The range between the minimal and maximal speed values that larvae can achieve is not drastically changing between different age groups (Figures S1G and S1H), suggesting that the physiological requirements for both inactive and fast swimming modes are given throughout the development of the animals. Consequently, the ability or the drive to swim must be actively regulated to explain our observations. Therefore, to understand why the animals display different modes of activity with distinct ages, we looked more closely at the distribution of the activity (total distance) over the time of our 5 min experiment and discovered that the largest shifts were observed during the first 60 s of our recordings (Figures 1K and 1L; Table S6). This time point corresponds to the arousal phase that is caused by the mechanical handling of the animals or the transfer to a novel environment, a common feature that has been reported before both in *Nematostella*^8^ and other organisms.^30^ This peak in arousal of larvae at 96 hpf coincides with the highest expression of putative sensory receptor proteins, such as TRP channels, some of which have been implicated in mechanosensitive behavior in *Nematostella vectensis*^19^^,^^31^ (Figures S1K and S1L), suggesting that the animals are more sensitive to external stimuli at this age and that this sensory information could be used to activate the swimming mode.

### Cilia change in length but not basal beating frequency

**Figure 2:**
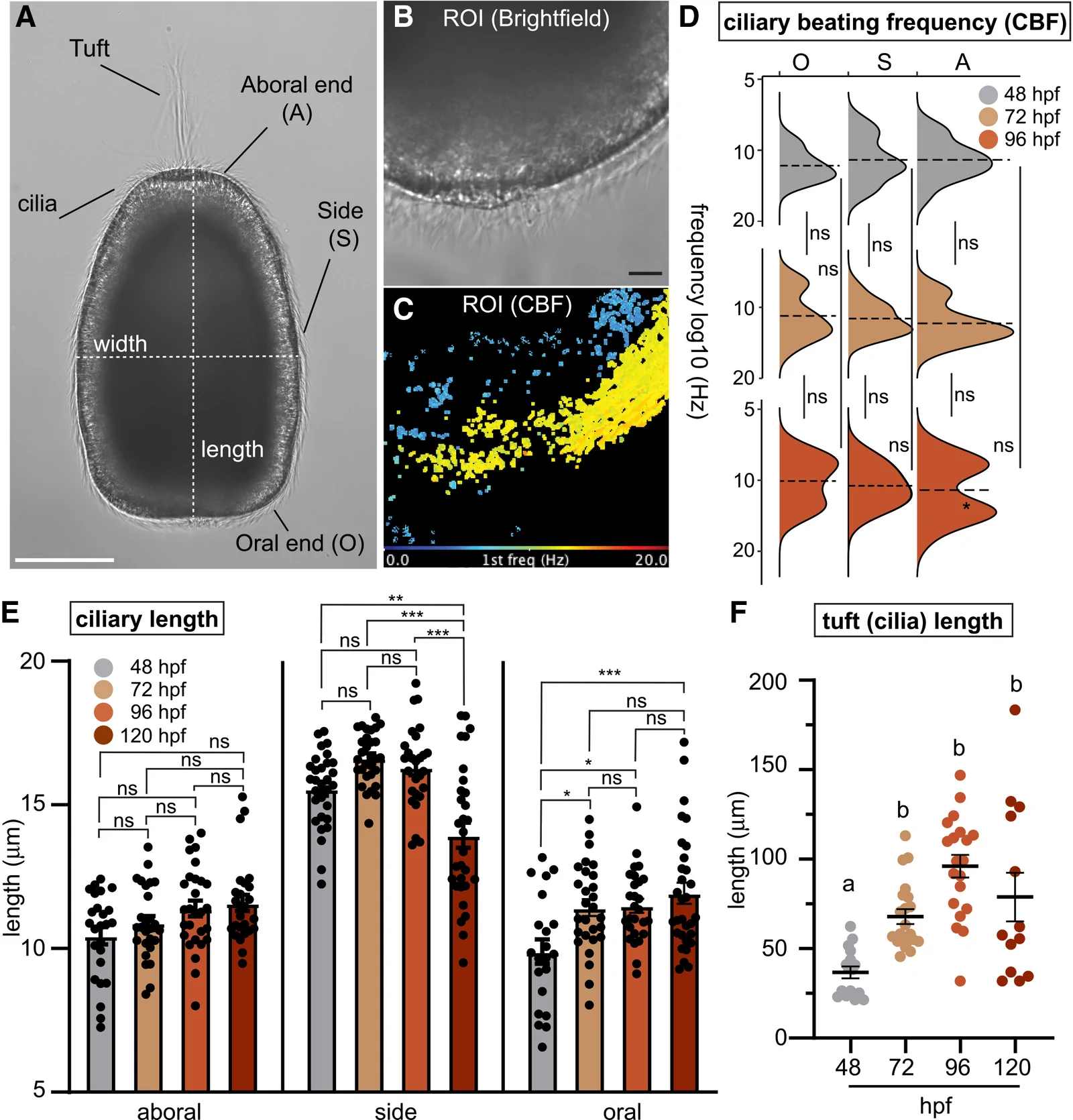
Cilia beating frequency and length during development (A) Representative image of a 96 hpf larva at 20× with regions of measurement denoted; scale bar,100 μm. (B) Image of region of interest (ROI) taken from a high-speed microscopy video taken for CBF analysis, scale bar, 10 μm. (C) Frequency map depicting peak frequency for each pixel. (D) Cilia beat at frequencies between 7 and 18 Hz with no significant differences (48–96 hpf *n* = 10–18, *N* = 6). Dashed line represents the means and letters denote statistical significance of ns *p* > 0.05 by the Kolmogorov-Smirnov test; asterisk (∗) denotes a significant deviation from unimodality by Hartigan’s Dip test. (E) Cilia at the aboral region of the body do not significantly change in length with age, whereas cilia along the side of the body decrease in length after 96 hpf and cilia at the oral end increase in length after 48 hpf (48–96 hpf *n* = 21–30, *N* = 5; 120 hpf *n* = 28–31, *N* = 1). Lines at mean ± SEM. Stars denote statistical significance of ns *p* > 0.05, ∗*p* < 0.05, ∗∗*p* < 0.01, and ∗∗∗*p* < 0.001 by Šídák’s multiple comparisons. (F) Tuft (cilia) length shows a trend of increasing length after 48 hpf (48–96 hpf *n* = 16–21, *N* = 5; 120 hpf *n* = 13, *N* = 1). Lines at mean ± SEM. Letters denote statistically significant differences in means of *p* < 0.05 by Dunn’s multiple comparisons (tuft cilia length). See also Tables S15–S17 for more information.

Cnidarian larvae are equipped with motile cilia that propel them through the water. Since the animal’s activity is changing over time, we suspected this might be mediated through cilia-related changes. Along this line of thought, we next investigated which aspects of larval ciliation are changing over the course of development. In cnidarian planula larvae, cilia act both as sensory and motor units and can most likely modify swimming patterns and behavioral complexity through both functions.^17^^,^^32^^,^^33^ We therefore investigated the overall length of the epithelial ciliation in the aboral, side, and oral region, (Figures 2A and 2E) as well as their beating frequency (Figures 2B–2D) and the length of the sensory organ cilia, the tuft (Figure 2F), to understand more about how ciliary details might contribute to the enhanced sensitivity and better steering abilities in older larvae.

Our measurements in restrained larvae show that the ciliary beating frequency (CBF) ranges between 7 and 18 Hz for all ages and body regions (Figures 2D and S2A), with no significant differences between the means. This observation shows that there is no overall increase in beating frequency as the animals age, and that larvae at all ages might be able to actively modify the CBF between set boundaries. Larvae at 96 hpf show a bimodal distribution of CBF in the aboral region (Table S15). It is possible that this sharp distribution points to an enhanced control over the beating at this age. Our setup does, however, not permit statements about CBF changes during swimming behavior, which might be more relevant to determine its effect on the speed in freely moving animals.

Other aspects of ciliation, such as increased ciliary length, might correspond to a larger power stroke when beating, increasing the force generated to propel the larval body through the water. We measured the length of cilia across the larval body over development (48–120 hpf), and when comparing average values for the three regions within the same age we found that cilia in the side region are significantly longer (range of means 13.94–16.63 μm) than those in the aboral (10.45–11.58 μm) and oral (9.89–11.93 μm) regions for all ages (Table S16). When comparing ciliary length within the same region across different ages, we find three primary features (Figure 2E). Firstly, there are no significant differences in apical ciliary length across ages. Secondly, 120 hpf have significantly shorter side cilia compared to 48–96 hpf (mean 48 hpf: 15.55 μm, 72 hpf: 16.62 μm, 96 hpf: 16.31 μm, 120 hpf: 13.94 μm), hinting toward a possible role in locomotion of this subgroup of cilia. Finally, 72–120 hpf larvae have significantly longer oral cilia compared to freshly hatched 48 hpf larvae (mean 48 hpf: 9.89 μm, 72 hpf: 11.42 μm, 96 hpf: 11.49 μm, 120 hpf: 11.93 μm), possibly in preparation for a more prominent role of the oral end in feeding in the metamorphosed animals (Figure 2E).

In addition, we measured the length of the cilia comprising the tuft of the apical organ and found significantly longer tuft cilia after 48 hpf (Figure 2F, mean 48 hpf: 36.81 μm, 72 hpf: 67.91 μm, 96 hpf: 96.07 μm, 120 hpf: 78.86 μm). Longer cilia increase both the physical reach and can house a greater number of receptors due to a larger membrane surface, leading to enhanced sensitivity for sensory cues, providing better spatial information that could facilitate more agile or faster swimming behavior.^34^^,^^35^ This is especially important considering the putative sensory role of the specialized cilia of the apical tuft. As the tuft cilia lengthen, not only does the larva experience improved sensory capability, but also enhanced polarity. A sensory role of motile cilia along the side of the body would further increase environmental perception to the whole body of the animal, leading to better flow sensing and improved proprioception, thereby facilitating complex swimming behaviors.^36^^,^^37^ At 120 hpf, apical tuft cilia show a wide spread of overall lengths and high variability (SEM of 13.55 μm, more than double that of any other age) (Figure 2F; Table S17), indicating the onset of the loss of the apical organ as previously reported^21^ and indicative of a decreasing need for this sensory structure when the larvae become non-motile. The timing of the loss of this structure coincides with the loss of motility but occurs before settlement. This reinforces the notion that the apical tuft is required for processing sensory information related to dispersal and swimming behavior, rather than settlement, and possibly forms a structure that aids in steering.^18^^,^^38^

### Developmental body shape changes correlate with locomotion

**Figure 3:**
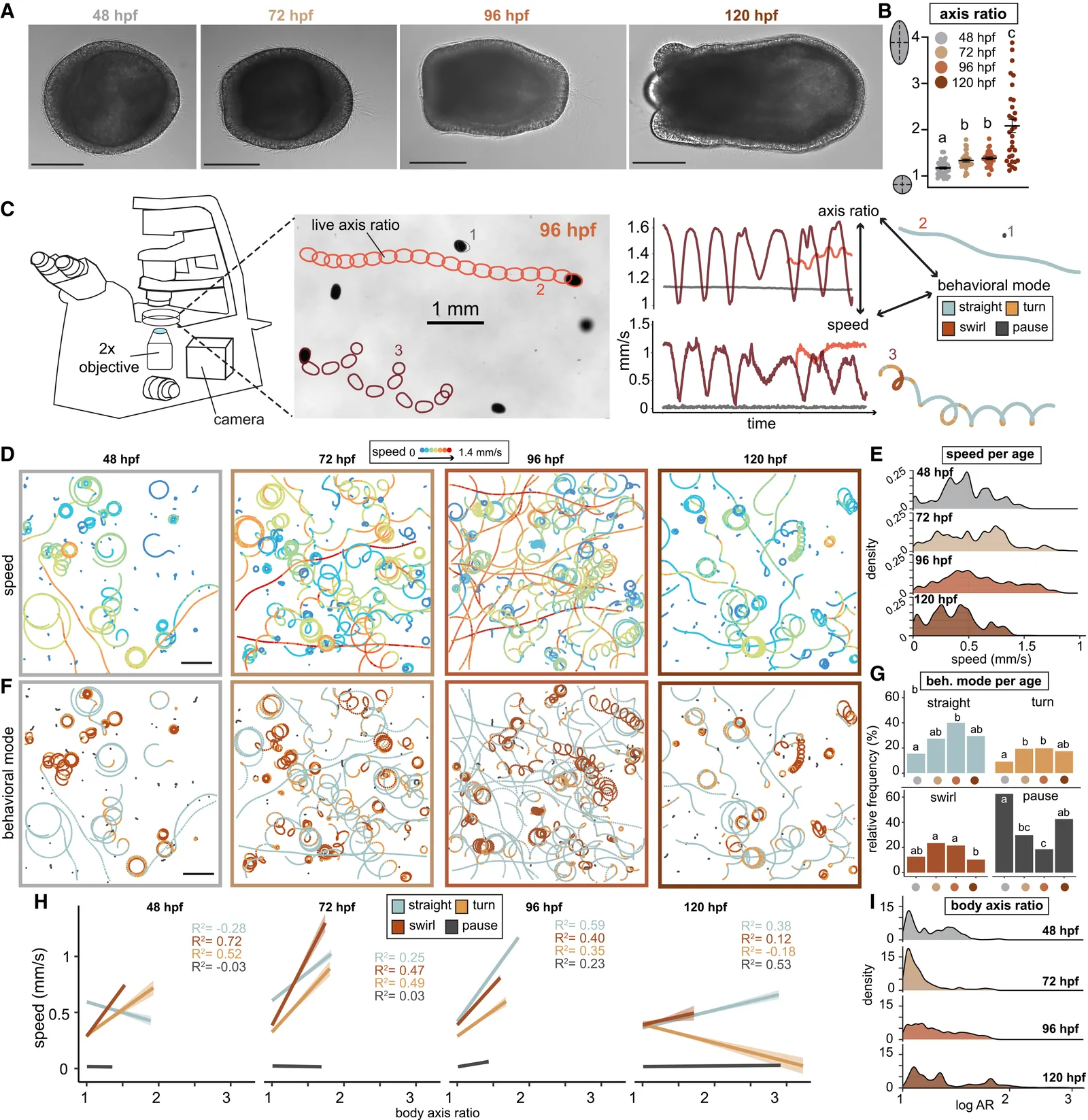
Body shape changes during development and locomotion behavior (A) Representative images of larvae over the course of development at 48, 72, 96, and 120 hpf at 20×, scale bars, 100 μm. (B) Body axis ratio of larvae at rest significantly increases with age (48–96 hpf *n* = 31–37, *N* = 4; 120 hpf *n* = 31, *N* = 1). Lines at mean ± SEM; letters denote statistically significant differences in the means by Dunnett’s T3 multiple comparisons. See also Table S20. (C) View of freely swimming larvae through the 2× objective in microscopy assay (Videos S1 and S2), allowing for live body size measurements while observing the swimming behavior. Speed and body axis ratio correlate in example larvae 1, 2, and 3. A behavioral mode analysis can be applied to downsampled swimming traces, categorizing moments within a trace as either straight, swirl, turn, and pause. See STAR Methods for more information. (D) Swimming traces for 48 hpf (*n* = 70, *N* = 3), 72 hpf (*n* = 80, *N* = 3), 96 hpf (*n* = 84, *N* = 2) and 120 hpf (*n* = 80, *N* = 3) colored based on the momentary speed; scale bar, 1 mm. (E) Density plots displaying the distribution of swimming speeds per age in the resampled dataset. (F) The resampled swimming traces for 48 hpf (*n* = 70, *N* = 3), 72 hpf (*n* = 80, *N* = 3), 96 hpf (*n* = 84, *N* = 2), and 120 hpf (*n* = 80, *N* = 3) colored based on the momentary behavioral mode, scale bar, 1 mm. (G) Bar graphs show the relative percentage of behavioral modes per age in the resampled dataset. Different letters denote statistically different means of *p* < 0.05 by Dunn’s multiple comparisons test. For more information, see Table S21. (H) Linear regression lines with 95% confidence intervals, and Spearman’s Rank correlation coefficients between the momentary body axis ratio and momentary speed for each behavioral mode, in all ages in the resampled dataset (48 hpf: straight slope = −190.2, turn slope = 456.6, swirl slope = 868.6, pause slope = −8; 72 hpf: straight slope = 508.1, turn slope = 706.5, swirl slope = 1241, pause slope = −10.5; 96 hpf: straight slope = 885, turn slope = 459.4, swirl slope = 707.5, pause slope = 106.1; 120 hpf: straight slope = 156.8, turn slope = −166.6, swirl slope = 144.6, pause slope = 6.1. See also Table S23). (I) Density plots display the distribution of body axis ratios observed for each age in the resampled dataset. X axis is on the log10 scale.

Given these observations, we wondered which other aspects of the larval body are changing over the course of development that would enable the animals to swim more linearly and therefore disperse faster and more efficiently when agitated. During our experiments, we frequently noticed that animals performed “shape shifts”. To investigate how the body shape is changing over time, we first measured it in immobilized animals (Figures 3A and 3B). Anatomical elongation can be quantified using the body axis ratio, a measurement where higher ratios between the length and the width indicate a more elongated, slender shape. Indeed, this body axis ratio was found to increase significantly with age (Figure 3B; mean ratio 48 hpf: 1.172, 72 hpf: 1.333, 96 hpf: 1.379, and 120 hpf: 2.082; Table S20).

Since it has been reported that cnidarian larvae routinely perform body shape shifts during active behavior,^6^^,^^9^ we wanted to investigate whether muscular contractions dynamically change the body axis ratio during locomotor behavior and if such contractions could be connected to the behavior of the animal. To answer these questions, we developed an assay with sufficient resolution to observe the animal’s swimming behavior concomitantly with body shape changes, using a microscopy-based observation arena (Figure 3C; see example larvae 1, 2, and 3, see also Videos S1 and S2). By integrating basic tracking metrics with information regarding local confinement and turn angles, we systematically classified four behavioral modes in larval swimming traces: “straight”, “swirl”, “turn”, and “pause”. In our assay, body shape, speed, and behavioral mode can then be assigned to the same coordinate, enabling the simultaneous analysis of three parameters for each individual (Figure 3C, right image, for details, see STAR Methods section).

Video S1. Example video from 2× microscopy in Figure 3 Larvae at 96 h postfertilization swim freely under the 2× objective.

Video S2. Example video of body measurements on a binary version of Video S1, related to Figure 3 A binary version of larvae aged 96 h postfertilization swimming under the 2× objective while taking body size measurements.

In agreement with data from the larger arena, swimming activity and speed are changing over development (Figures 3D and 3E). Additionally, the relative frequency of certain behavioral modes per age is changing when quantified by relative occurrence per track (Figures 3F and 3G). Planula larvae (48–96 hpf) are increasingly swimming “straight” and pause less before these values increase again after metamorphosis (120 hpf) (Figure 3G straight: 48 hpf 16%, 72 hpf 27%, 96 hpf 40%, 120 hpf 30%; Turn: 48 hpf 9%, 72 hpf 19%, 96 hpf 20%, 120 hpf 18%; Swirl: 48 hpf 13%, 72 hpf 23%, 96 hpf 22%, 120 hpf 10%; Pause: 48 hpf 63%, 72 hpf 30%, 96 hpf 19%, 120 hpf 43%. See also Table S21). Locally confined “swirling”, and reorientation behavior such as “turning”, might indicate heightened environmental sampling in search of sensory information. These modes occur more frequently in late planulae (72 hpf: 23% swirl, 19% turn; 96 hpf: 22% swirl, 20% turn), coinciding with increased TRP channel expression and other sensory behaviors as described in Figure 1L.

As both body axis ratio and locomotor patterns are changing over development (Figures 3E–3G and 3I), we were curious as to whether there is a correlative relationship between momentary elongation and momentary swimming speed during each of the behavioral modes (Figure 3H; Table S22). Indeed, longer animals swim, on average, faster during behavioral modes such as “turn”, “swirl” and “straight”, as indicated by the positive correlation in 72 hpf and 96 hpf (straight: 72 hpf *R*^2^ = 0.25, slope = 508.1; 96 hpf *R*^2^ = 0.59, slope = 885; turn: 72 hpf *R*^2^ = 0.49, slope = 706.5; 96 hpf *R*^2^ = 0.35, slope = 459.4; Swirl: 72 hpf *R*^2^ = 0.47, slope = 1241; 96 hpf *R*^2^ = 0.40, slope = 707.5). Interestingly, 48 hpf and metamorphosed (120 hpf) larvae did not swim at high speeds while swimming “straight”. In fact, there is a slight negative relationship between body elongation and speed in 48 hpf (*R*^2^ = −0.28, slope = -190.2) in this mode, and a less positive relationship in 120 hpf (*R*^2^ = 0.38, slope = 156.8) compared to 96 hpf. The correlation between body axis ratio and swimming speed during straight swimming is increasing until a maximum value is reached at 96 hpf. Together with the higher frequency of straight swimming behavior, we here find a major determinant of the larvae’s increase in dispersal efficiency at this age: animals coordinate body axis ratio and speed to optimize dispersal through enhanced straightness of the swimming trajectory.

These observations are not just due to larger larvae swimming with greater ease. The total size of the larvae (measured as area) is neither correlated with speed, nor with elongation (Figures S3A and S3B; Tables S23 and S24), showing that the specific body shape is contributing to increased swimming speeds in the larval stage. To our surprise, the body area (animal size) seems to be a determining factor for locomotion, as animals that reach a size threshold of around 60 mm^2^ (Figure S3B, dashed line) move quite slowly as compared to their smaller siblings. Whether size is a determining factor for metamorphosis or locomotion will have to be determined in future experiments.

How exactly shape changes of the larvae influence their swimming behavior and efficiency is unclear. One possibility is that the ciliary tuft of the apical organ is the reason for the elongation of the aboral area. We observed that larvae in straight and swirling behavior often show some constriction toward the apical tuft and that the tuft is bundled and pointing straight ahead (Figure S3C). This could enhance the polarity of the larvae either for better steering if the tuft serves a paddle function as suggested in^38^ or for further sensory reach.

### Cilia and neurons play distinct roles in swimming behavior

**Figure 4:**
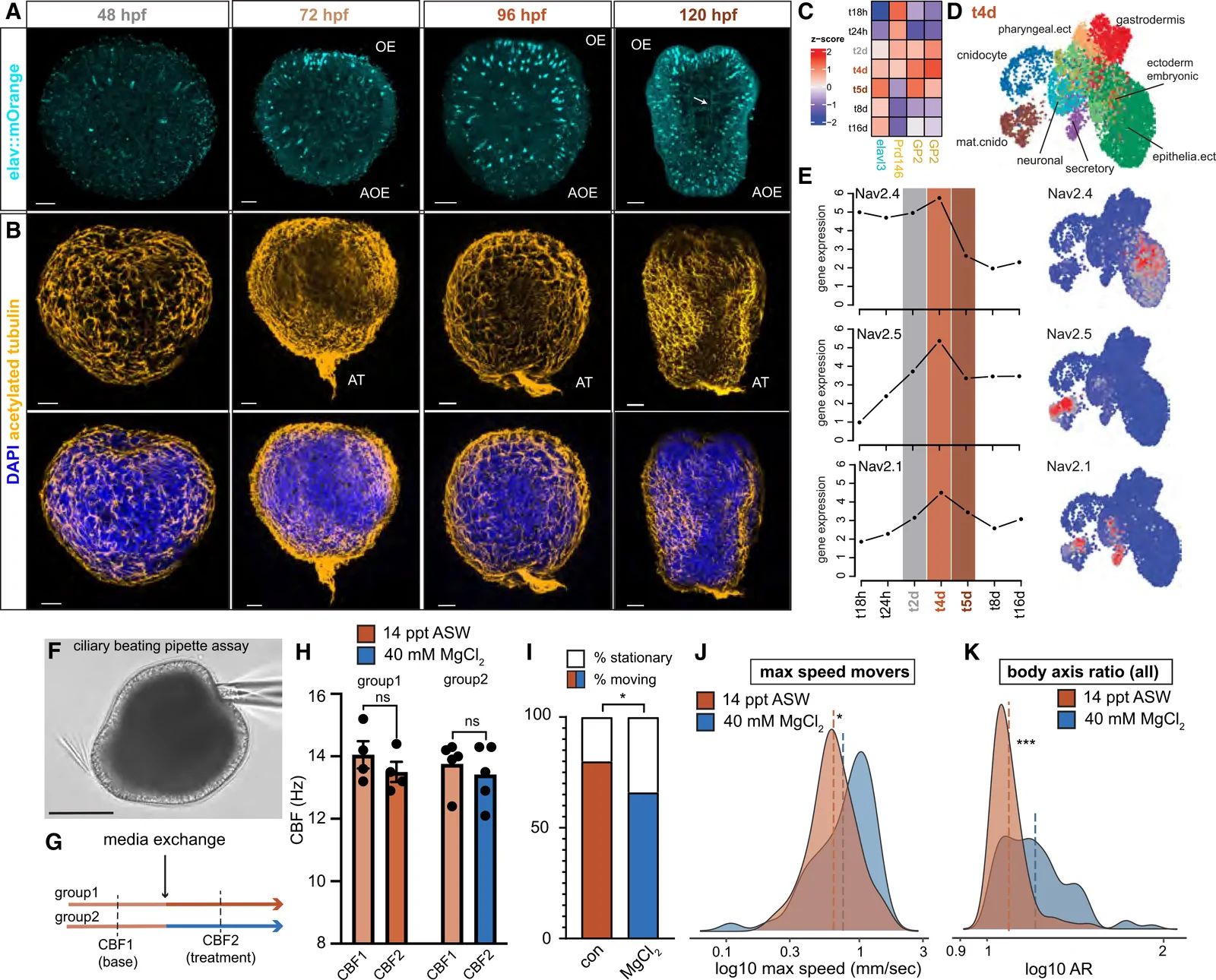
Nervous system and cilia are distinct subsystems involved in swimming behavior (A) *Elav::mOrange* larvae stained with dsRed between 48 and 120 hpf. Arrow indicates nerve tracks; scale bars, 20 μm. (B) Ciliation of larvae between 48 and 120 hpf visualized using acetylated tubulin (yellow) and DAPI for nuclear stain (dark blue), (OE = oral end, AOE = aboral end, AT = apical tuft, scale bars, 20 μm). (C) Expression profiles of marker genes. Data show relative average expression values across developmental time. (D) Two-dimensional representation of single-cell transcriptional landscape at age td4 (∼96 hpf). Data points represent single cells labeled by cell groups reported in ref.^39^ (E) Developmental average expression profiles (left) and 2D projection of single-cell expression values (right) of genes encoding Nav ion channels. (F) Representative image of a 96 hpf pipette-attached larva at 20×, scale bars, 100 μm. (G) Schematic of pipette-assay protocol with different perfused treatments as indicated by arrow and “media exchange”. (H) Comparison of CBFs at CBF 1 and CBF 2 video timepoints revealed no significant differences when exposed to the control ASW condition (*n* = 4, *N* = 2) or the MgCl_2_ treatment condition (*n* = 5, *N* = 2) for 5 min, where ns *p* > 0.05 by Wilcoxon matched pairs signed rank test. Lines at mean ± SEM. (I) Ratios of moving and stationary larvae for the control (*n* = 84, *N* = 3, 80% moving) and MgCl_2_-treated (*n* = 88, *N* = 3, 66% moving) larvae. Two-tailed two-proportion *Z* test ∗*p* = 0.039. (J) Distributions of maximum speeds between the control (*n* = 67, *N* = 3) and MgCl_2_ treated (*n* = 58, *N* = 3) larvae (moving animals only). Line at the mean, ∗∗*p* = 0.0016 by Kolmogorov-Smirnov test. (K) Distributions of axis ratios between the control (*n* = 75, *N* = 3) and MgCl_2_ treated (*n* = 84, *N* = 3) larvae. Line at the mean, ∗∗∗*p* < 0.0001 by the Kolmogorov-Smirnov test. See also Tables S25–S29.

Since the function of the larval cilia (particularly the apical tuft), the nervous system, and their interplay in locomotion remain poorly understood in *Nematostella* larvae, we were keen on describing these structures concomitantly. By using the same timepoints as the behavioral experiments, we hoped to deduce how anatomical differences correlate with the observed behavioral changes. To this end, we performed immunohistochemistry of larvae aged 48 hpf–120 hpf with anti-DsRed in the transgenic *Elav::mOrange* line that selectively labels neurons (Figure 4A)^40^ and with anti-acetylated tubulin to show the ciliary structures (Figure 4B). In addition to the histology, we re-analyzed single-cell transcriptomic data at relevant developmental stages^39^ to understand how cellular and molecular components endow the animal with distinct functional subsystems that enable a more controlled swimming pattern over time (Figures 4C–4E, S4B, and S4C).

We found that the larvae show an increasing number of neurons (Figure S4A), as previously reported^41^^,^^42^ with a small number of ectodermal sensory neurons in the early planula stage and larger numbers in both ecto- and endoderm in the late planula. Finally, clearly visible nerve tracks are found by the time animals are developing prominent tentacle buds (Figure 4A, arrow). Remarkably, we observed that the largest number of sensory neurons in the aboral area appears only in the 120 hpf stage (Figure 4A, right), suggesting that older larvae have an increased need for sampling substrates. *Nematostella* larvae indeed metamorphose before they settle and can reattach for a long period of the early polyp stage.^8^ In agreement with the literature and our measurements (Figure 2F), the 48 hpf larvae rarely possess an apical tuft. This structure appears later and remains prominent up until 5dpf, when it becomes less dense, and some larvae were observed to have already lost the tuft. Interestingly, when looking in the single-cell dataset from^39^ using markers for neurons (elavl3), the apical organ (Prd146) and the ectodermal epithelium (GP2) from,^21^ we found that, while the neuronal marker is increasing in agreement with our immunohistological observations, both the apical organ ciliated cells (Prd146) and some ectodermal cells labeled with GP2 seem to be largely constrained to the motile phase of the larvae (Figures 4C, 4D, and S4B). These observations are consistent with the expression of Nav2.4 as a proxy for the functional capacity of the ciliated epithelial cells. Nav2.4 is strongly expressed in younger larval ages and disappears almost completely after the animals seize swimming activity (Figure 4E). This sodium channel is exclusively found in the larval ectoderm and apical organ cells^43^ (Figure 4E right image), suggestive of a role in excitability in these ectodermal cells (e.g., ciliary beating, contractility). In contrast to Nav2.4, strong expression of Nav2.1 and 2.5, sodium channels that are characteristic of other excitable cells, such as neurons and cnidocytes, is observed only in later larval stages. These cellular and molecular data suggest that the larvae lose molecular and potentially also cellular components associated with ciliary locomotion. Altogether, these observations make it likely that individual systems play a role at different stages in the animal’s life and, in addition, serve distinct functions with respect to ontogenetically restricted behaviors.

To further test this hypothesis, we blocked synaptic transmission using magnesium chloride (MgCl_2_)^44^ and observed how the inhibition of the nervous system affects ciliary beating and swimming behavior. We used 96 hpf larvae, the motile stage with the most developed nervous system, most prominent apical tuft, and highest sensitivity to sensory stimuli. Baseline ciliary beating as measured in larvae fixed by a pipette (Figure 4F) did not change when MgCl_2_ was applied as compared with control animals, which remained in normal media (Figures 4G and 4H). Cilia in the larvae, therefore, beat without neuronal input and are, at least during basal conditions, not influenced by neuronal activity. Ciliated cells might create their own sensory-motor units, regulating beating frequency intrinsically in response to external information, such as mechanical or light stimuli, rather than being influenced by neuronal input. This finding is supported by previous observations of sensory receptors being expressed directly in epithelial cells in *Nematostella* planula larvae^19^^,^^32^ and by our expression analysis, which finds a large proportion of the highly expressed TRP channels at 96 hpf in epithelial cells (Figure S4C).

When MgCl_2_ was applied to freely moving animals, we observed that, despite the lack of an obvious change in CBF, fewer animals were among the actively swimming proportion (66% moving) compared to the control (80% moving) (Figure 4I, for the definition of moving vs. stationary, see STAR Methods). While the reduction of motile animals was significant, the majority of the planula larvae remained activated through arousal by handling. This result strengthens the theory of cilia as independent sensory-motor units and shows that a fully functioning nervous system is not basally required for swimming but rather constitutes an additional layer of locomotor control.

To further investigate what this role could be, we looked more carefully at the swimming and body shape differences between control and magnesium-treated animals. Magnesium-treated animals showed substantial changes in both parameters (Figures 4J and 4K). Actively swimming larvae (movers only) (Figures 4J and S4D) swam faster (median mean speed 0.88 mm/s) as compared to the control (median mean speed 0.63 mm/s). When plotting the distribution of the speed quartiles of all animals (Figure S4E), we found that magnesium-treated animals were essentially locked in two speed modes: either stationary (Q1) or at high speed (Q4), and only a few larvae were found in the intermediate speed modes.

*Nematostella* larvae do not show changes in the range between maximum and minimum speed, but rather appear to acquire the ability to utilize their body more efficiently over developmental time. Locomotion gait is mediated by matching internal and external sensory information to reach optimal swimming patterns. Therefore, larval swimming gait would include proprioceptive responses and appropriate adjustment of the body shape to a given external sensory environment. Indeed, when plotting the axis ratio and mean speed of the animals, it became evident that the inhibition of synaptic transmission leads to a disconnect of these parameters (Figure S4F). In particular, the relationship between speed, body axis ratio, and behavioral mode showed that neuronal activity is: 1. important for the positive correlation of these parameters in “straight” and “turn” behaviors in particular and 2. independent from the value of the axis ratio (swirl and pause slope are not affected but AR is) (Figure S4G).

Swimming gait is important for maximal locomotor efficiency and endows the animal with the capacity to fine-tune its behavior in agreement with external and internally collected sensory information, a process that seems to rely on functional synaptic transmission. Reorganizing the body shape through muscular contractions can influence ciliary orientation and consequently lead to speed modifications induced by flow field changes. These changes will in turn influence proprioceptive signaling, helping the animal to assess its behavior and orient itself with respect to the environment.^37^

## Discussion

In the present study, we report, for the first time, detailed aspects of swimming behavior of the *Nematostella vectensis* planula larvae across systematically chosen developmental timepoints and examine its relationship to concomitantly appearing cellular and molecular features. Larval behavior is changing over time, leading to optimized dispersal through the integration of external and internal sensory information that enables the animal to utilize their form in a more efficient way. While some aspects remain relatively stable over development, such as minimum and maximum speed, length of motile cilia, and their beating frequency, the larvae instead shift on the scale of possibilities toward more linear trajectories and faster speeds through the polarization and sharpening of sensory functions. The advance of the sensory systems allows animals to be more active and “sensitive” in response to external stimuli such as light and mechanical stimuli. Additionally, a reafference system based on neuronally mediated body shape contractions is optimized for higher speed and more linear swimming. This sensory integration makes *Nematostella* larvae versatile swimmers with a rich repertoire of behavioral modes.

For an animal with the size of the *Nematostella* larvae, swimming in aqueous medium is energetically extremely costly. Indeed, ciliated epithelial cells are metabolically highly active,^33^ and the ciliary length might be perfectly adapted to swimming efficiency in small organisms.^45^ Given that a helical swimming pattern is stereotyped across several marine species, some aspects of swimming are potentially also confined by physical laws that determine the maximal efficiency of movement and optimize flow fields.^15^^,^^27^^,^^46^^,^^47^ While *Nematostella* swimming speed (0-3 mm/s in still water) and its body size may not endow the animal with the capacity to resist larger currents, their swimming ability is well adapted to facilitate dispersal and habitat selection within their natural ecological niche of tidal pools and marshes, where water may remain still or experience incremental increases. Responding to sensory stimuli by increasing both activity and fine-tuning behavior, reflected in the ability to control swimming modes, facilitates dispersal and enables the larvae to invest their efforts with maximal efficiency. Higher activity in larvae has been observed after mechanical stimulation or in response to short wavelengths of light.^11^^,^^30^ In contrast, responses to stimuli can also induce the opposite response, including drastic body contraction and the cessation of various types of activity.^6^^,^^13^ Constraining dispersal time both during development and also acutely, as observed for *Nematostella* planula larvae, is an adaptive behavior for an anemone that lives in a relatively restricted area,^48^ where large dispersal distances could lead to a loss of surviving offspring to the vast ocean currents.

Furthermore, sensory perception will also include self-sensing in the form of proprioception and flow sensing, leading to optimization in swimming gait.^37^ The ability to actively and effectively utilize the set of swimming modes available to the animals, and to transition between those modes at appropriate timepoints, seems to be governed by an intrinsic mechanism that “turns on” at a certain developmental stage. This increase in sensory abilities co-occurs with increasing numbers of TRP channels in epithelial cells and concomitant elongation of the apical tuft. Being able to access the external and internal sensory information allows the animal to be activated through appropriate stimulation and to optimize swimming gait to reach their goals in the ontogenetically available time.

How cnidarian larvae sense their environment is not well understood. Recent investigations into the cellular and molecular architecture of different cnidarian planulae have, however, opened the possibility for detailed investigations of such aspects.^20^^,^^21^^,^^39^^,^^49^ In agreement with previous observations that have shown that the apical organ is not required for the larvae-polyp transition^21^^,^^22^ and is lost in some animals prior to settlement,^18^ our data suggest a sensory role of the apical tuft mainly in swimming behavior. Larvae display larger activation through mechanical input and more linear trajectories at ages that show a prominent tuft, while, at competency, when most animals begin the search for a suitable habitat, the tuft is degrading or lost. Several studies have found sensory receptors expressed in the apical organ^19^^,^^32^ and, together with reports documenting the apical tuft to move as a unit during swimming with a sweeping movement,^17^^,^^18^ it is likely to possess a tactile-sensory or stirring purpose, providing clear polarization of the elongated planula larvae that creates a sensory directionality to guide swimming movement. The increase in tuft length with age might further increase sensory perception by allowing more receptors to be housed in an individual cilium, which would increase the sensitivity of this organ.^50^ Which other sensory modalities *Nematostella* larvae use and how the ciliated cells communicate among each other and with the nervous system will be of great interest in future investigations.

In addition to the tuft cilia, our analysis reveals that larvae might not just lose the tuft, but possibly a larger fraction of sensory-motile cilia that enable swimming behavior in the planula larvae. These epithelial cells show expression of functional ion channels only during the motile stage of the larvae, such as Nav2.4 and TRP channels, strongly suggesting that these cells might serve a sensory-motor purpose too. Indeed, previous reports have found sensory-motor cells in cnidarian planula larvae,^33^ and it is well known that most cilia can sense external stimuli. How and if these ciliated cells and their cognate molecular machinery are involved in the larvae’s swimming is unclear at this time.

A second aspect of improved swimming control is the development of the nervous system. When and how the first nervous systems appeared, and what their role was, is under much active debate. It has been suggested that the nervous system in ciliated metazoan larvae first appeared for signal amplification and direct sensory motor connections.^51^ We find that many sensory receptors and other “neuronal” ion channels such as voltage-gated sodium channels are expressed in the ciliated epithelium, possibly in some form of larval-specific sensory-motor unit. This ciliated sensory-motor unit appears to be in large parts independent of the nervous system, since blocking synaptic transmission only partially impairs swimming and mostly affects the fine-tuning of behaviors that are mediated by muscular contractions. In other cnidarian larvae, it has been observed that the nervous system is absent or rudimentary and that ciliary sensory motor units might be the only means of steering the animal,^33^ a feature that is maintained from more basal ciliated larvae.^51^

We suggest here that the function of the nervous system is the optimized orientation of the sensory organ and possibly other ciliary structures, such as the swimming cilia, which may be controlled by muscular contractions and relaxations. Such active reorientation of the ciliated epithelium might be used to position ciliary rows along the proper body axes, producing more efficient thrust irrespective of CBF. Previous studies have shown that body shape and ciliation can be modified for dispersal efficiency^52^ and that the generation of coherently directed flow requires cilia to be optimally oriented.^53^ The body shape and the resulting sensory experience influence the swimming ability and connect appropriate speeds with specialized behaviors. Shape-shifting has been reported in several cnidarian larvae,^6^^,^^9^^,^^54^ and further analysis might shed more light on how exactly the complex body shape patterns observed here serve specialized functions in the larva.

### Limitations to the study

In the present study, we systematically characterized the swimming behavior of the planula larvae as the animals develop, to describe some key aspects of their behavior. Future studies should venture into cellular and molecular aspects of physiology and sensory perception as well as other types of behaviors. Our study contains some caveats, specifically with respect to the subcellular physiology of cnidarians, which is not well described. How and if different subsystems (muscle, cilia, and neurons) exactly work in concert to optimize signal perception and processing to produce optimal locomotion is likely complex and cannot fully be solved with a generic channel blocker such as MgCl_2_. Targeted manipulation will require in-depth study of cellular physiology and molecular pharmacology.

Furthermore, the behavioral assays used in our study contain caveats and limitations. In the microscopy behavior, for example, it is difficult to determine exact speed and axis ratio when larvae turn in the vertical plane, and it would of course be optimal to film the animal in all dimensions to avoid data loss. Irrespective of these limitations, our study provides important insights into sensory and motor behavior in cnidarian larvae and provides a basis on which to build more detailed knowledge with targeted manipulations.

Comparative studies can advance our knowledge on how the planula larvae disperse and subsequently find a permanent settlement site with respect to their ecosystem and its physical characteristics. Understanding such details of this critical phase in the larva’s life is important to solve questions related to the current climate crisis and loss of biodiversity. To protect ecosystems, we must understand them. *Nematostella vectensis* has served as a pioneering model in the biology of cnidarians, and it is urgently required that we develop more tools to understand the fundamental aspects of cnidarian biology.

## Resource availability

### Lead contact

Further information and requests for resources should be directed to the lead contact: lena.van.giesen@ntnu.no.

### Materials availability

This study did not generate new materials.

### Data and code availability

• Data: Data for *Nematostella vectensis* (48–120 hpf) body size and coordinate information (related to Figure 3) can be found at Figshare and are publicly available as of the date of publication at https://doi.org/10.6084/m9.figshare.32820290. All other data reported in this paper will be shared by the lead contact upon request.
• Code: Original code can be found at Figshare and is publicly available as of the date of publication https://doi.org/10.6084/m9.figshare.32817278.
• Other: Accession numbers from previously described genes analyzed in Figure 4, are listed in the key resources table. Any additional information required to reanalyze the data reported in this paper are available from the lead contact upon request.

## Acknowledgments

We thank Fabain Rentzsch (UiB) for gifting the WT and *Elav1::mOrange* animals and members of the van Giesen Lab for help with *Nematostella* maintenance. We are grateful for feedback on the manuscript from members of the van Giesen Lab and Fabian Rentzsch. This research was supported by the ERC StG “EnvIronchannel” (101076516) granted to L.v.G.

## Author contributions

M.L. performed behavioral analysis, microscopy, and immunohistochemistry; M.M.S. performed imaging experiments, ciliary beating, and body measurements. J.D.V. and J.H. performed computational analysis. All authors conceptualized the study, designed and created the figures, performed statistical analysis, and wrote the manuscript.

## Declaration of interests

The authors declare no competing interests.

## STAR★Methods

### Key resources table

**Table:** 

REAGENT or RESOURCE	SOURCE	IDENTIFIER
**Antibodies**		

Mouse Anti-Tubulin, Acetylated	Sigma-Aldrich (Merck)	Cat#T6793; RRID: AB_477585
Rabbit anti-DsRed (Living Colors)	Takara Bio	Cat#632496
Goat anti-Mouse Alexa Fluor 647	ThermoFisher Scientific	Cat# A-21240; RRID:AB_2535809
Goat anti-Mouse CY3	Abcam	Cat# AB97035

**Chemicals, peptides, and recombinant proteins**		

Coral Pro Salt	Red Sea Aquarium System	Cat# R11220
Magnesium chloride (MgCl2) hexahydrate	Sigma-Aldrich (Merck)	Cat# M2670
Magnesium chloride (MgCl_2_) solution	Sigma-Aldrich (Merck)	Cat# M1028
Paraformaldehyde (PFA)	Sigma-Aldrich (Merck)	Cat#158127
Dimethyl sulfoxide (DMSO)	Sigma-Aldrich (Merck)	Cat# D8418
Phospate buffered saline (PBS)	Sigma-Aldrich (Merck)	Cat# P4417
Hydrogen peroxide 30% unstabilised, AnalaR NORMAPUR® for trace analysis	WVR Chemicals	Cat# 23615.421
TritonTM X-100	Sigma-Aldrich (Merck)	Cat# X100
Normal Goat Serum	Sigma-Aldrich (Merck)	Cat# G9023
DAPI	ThermoFisher Scientific	Cat# D1306
ProLong™ Glass Antifade Mountant	ThermoFisher Scientific	Cat# P36980

**Deposited data**		

Nematostella vectensis 48 hpf–120 hpf body and coordinate information	This paper	https://doi.org/10.6084/m9.figshare.32820290

**Experimental models: Organisms/strains**		

*Nematostella vectensis* Wildtype	Fabian Rentzsch	N/A
*Nematostella vectensis* elav::mOrange	Fabian Rentzsch	N/A

**Software and algorithms**		

Arduino IDE 2.2.1	Arduino	N/A
Fiji ImageJ	Schindelin et al.^55^	N/A
TrackMate	Ershov et al.^56^	N/A
MultiStackReg	Thévenaz et al.^57^	N/A
FreQ	Jeong et al.^58^	N/A
GraphPad Prism	GraphPad software	www.graphpad.com
R Version 4.3.1 and 4.5.0		https://www.R-project.org/
Computational framework ACTIONet	Mohammadi et al.^59^	GitHub - shmohammadi86/ACTIONet at R-release · GitHub
HMMER software package for sequence analysis v3.4	Eddy^60^	(http://hmmer.org/)
dplyr	Hadley Wickham et al.	https://doi.org/10.32614/CRAN.package.dplyr
ggplot2	Hadley Wickham et al.	https://doi.org/10.32614/CRAN.package.ggplot2
ggridges	Claus O. Wilke	https://doi.org/10.32614/CRAN.package.ggridges
tidyr	Hadley Wickham et al.	https://doi.org/10.32614/CRAN.package.tidyr
purr	Hadley Wickham and Lionel Henry	https://doi.org/10.32614/CRAN.package.purrr
stringr	Hadley Wickham	https://doi.org/10.32614/CRAN.package.stringr
zoo	Achim Zeileis et al.	https://doi.org/10.32614/CRAN.package.zoo
data.table	Tyson Barrett et al.	https://doi.org/10.32614/CRAN.package.data.table
diptest	Martin Maechler	https://doi.org/10.32614/CRAN.package.diptest
broom	David Robinson et al.	https://doi.org/10.32614/CRAN.package.broom
emmeans	Russell V. Lenth et al.	https://doi.org/10.32614/CRAN.package.emmeans
Behavioral mode analysis *Nematostella vectensis* larval swimming (R code)	This paper	https://doi.org/10.6084/m9.figshare.32817278

**Other**		

8-RGB LED NeoPixel Sticks	Adafruit	Cat# 1426
Mega 2560 Rev3	Arduino	SKU# A000067
GUM ® Orthodontic Wax, unflavored	Sunstar	Cat# 723RQD
Borosilicate Glass with Filament	Sutter Instruments	Cat# BF150-86-10HP
0.2 μM PES Membrane Filtration Cup	VWR	Cat# 514-0340
Gene IDs related to Figure 4		
NV2.22582	Cole et al.^39^	repository [GSE200198 and GSE154105], https://www.ncbi.nlm.nih.gov/geo
NV2.24495	Cole et al.^39^	repository [GSE200198 and GSE154105], https://www.ncbi.nlm.nih.gov/geo
NV2.7365	Cole et al.^39^	repository [GSE200198 and GSE154105], https://www.ncbi.nlm.nih.gov/geo
NV2.8602	Cole et al.^39^	repository [GSE200198 and GSE154105], https://www.ncbi.nlm.nih.gov/geo
NV2.2930	Cole et al.^39^	repository [GSE200198 and GSE154105], https://www.ncbi.nlm.nih.gov/geo
NV2.6626	Cole et al.^39^	repository [GSE200198 and GSE154105], https://www.ncbi.nlm.nih.gov/geo
NV2.7252	Cole et al.^39^	repository [GSE200198 and GSE154105], https://www.ncbi.nlm.nih.gov/geo
NV2.18039	Cole et al.^39^	repository [GSE200198 and GSE154105], https://www.ncbi.nlm.nih.gov/geo
NV2.16727	Cole et al.^39^	repository [GSE200198 and GSE154105], https://www.ncbi.nlm.nih.gov/geo
NV2.15467	Cole et al.^39^	repository [GSE200198 and GSE154105], https://www.ncbi.nlm.nih.gov/geo
NV2.10225	Cole et al.^39^	repository [GSE200198 and GSE154105], https://www.ncbi.nlm.nih.gov/geo
NV2.15790	Cole et al.^39^	repository [GSE200198 and GSE154105], https://www.ncbi.nlm.nih.gov/geo
NV2.521	Cole et al.^39^	repository [GSE200198 and GSE154105], https://www.ncbi.nlm.nih.gov/geo
NV2.519	Cole et al.^39^	repository [GSE200198 and GSE154105], https://www.ncbi.nlm.nih.gov/geo
NV2.12744	Cole et al.^39^	repository [GSE200198 and GSE154105], https://www.ncbi.nlm.nih.gov/geo
NV2.23620	Cole et al.^39^	repository [GSE200198 and GSE154105], https://www.ncbi.nlm.nih.gov/geo
NV2.8726	Cole et al.^39^	repository [GSE200198 and GSE154105], https://www.ncbi.nlm.nih.gov/geo
NV2.8930	Cole et al.^39^	repository [GSE200198 and GSE154105], https://www.ncbi.nlm.nih.gov/geo
NV2.1902	Cole et al.^39^	repository [GSE200198 and GSE154105], https://www.ncbi.nlm.nih.gov/geo
NV2.1904	Cole et al.^39^	repository [GSE200198 and GSE154105], https://www.ncbi.nlm.nih.gov/geo
NV2.7219	Cole et al.^39^	repository [GSE200198 and GSE154105], https://www.ncbi.nlm.nih.gov/geo
NV2.16659	Cole et al.^39^	repository [GSE200198 and GSE154105], https://www.ncbi.nlm.nih.gov/geo
NV2.7555	Cole et al.^39^	repository [GSE200198 and GSE154105], https://www.ncbi.nlm.nih.gov/geo
NV2.20195	Cole et al.^39^	repository [GSE200198 and GSE154105], https://www.ncbi.nlm.nih.gov/geo
NV2.18254	Cole et al.^39^	repository [GSE200198 and GSE154105], https://www.ncbi.nlm.nih.gov/geo
NV2.21369	Cole et al.^39^	repository [GSE200198 and GSE154105], https://www.ncbi.nlm.nih.gov/geo
NV2.20496	Cole et al.^39^	repository [GSE200198 and GSE154105], https://www.ncbi.nlm.nih.gov/geo
NV2.18451	Cole et al.^39^	repository [GSE200198 and GSE154105], https://www.ncbi.nlm.nih.gov/geo
NV2.6894	Cole et al.^39^	repository [GSE200198 and GSE154105], https://www.ncbi.nlm.nih.gov/geo
NV2.18932	Cole et al.^39^	repository [GSE200198 and GSE154105], https://www.ncbi.nlm.nih.gov/geo
NV2.8942	Cole et al.^39^	repository [GSE200198 and GSE154105], https://www.ncbi.nlm.nih.gov/geo
NV2.8357	Cole et al.^39^	repository [GSE200198 and GSE154105], https://www.ncbi.nlm.nih.gov/geo
NV2.18388	Cole et al.^39^	repository [GSE200198 and GSE154105], https://www.ncbi.nlm.nih.gov/geo
NV2.2120	Cole et al.^39^	repository [GSE200198 and GSE154105], https://www.ncbi.nlm.nih.gov/geo
NV2.8911	Cole et al.^39^	repository [GSE200198 and GSE154105], https://www.ncbi.nlm.nih.gov/geo
NV2.8294	Cole et al.^39^	repository [GSE200198 and GSE154105], https://www.ncbi.nlm.nih.gov/geo
NV2.659	Cole et al.^39^	repository [GSE200198 and GSE154105], https://www.ncbi.nlm.nih.gov/geo
NV2.103	Cole et al.^39^	repository [GSE200198 and GSE154105], https://www.ncbi.nlm.nih.gov/geo
NV2.229	Cole et al.^39^	repository [GSE200198 and GSE154105], https://www.ncbi.nlm.nih.gov/geo
NV2.13263	Cole et al.^39^	repository [GSE200198 and GSE154105], https://www.ncbi.nlm.nih.gov/geo
NV2.642	Cole et al.^39^	repository [GSE200198 and GSE154105], https://www.ncbi.nlm.nih.gov/geo
NV2.9107	Cole et al.^39^	repository [GSE200198 and GSE154105], https://www.ncbi.nlm.nih.gov/geo
NV2.8359	Cole et al.^39^	repository [GSE200198 and GSE154105], https://www.ncbi.nlm.nih.gov/geo
NV2.7363	Cole et al.^39^	repository [GSE200198 and GSE154105], https://www.ncbi.nlm.nih.gov/geo
NV2.5171	Cole et al.^39^	repository [GSE200198 and GSE154105], https://www.ncbi.nlm.nih.gov/geo
NV2.3562	Cole et al.^39^	repository [GSE200198 and GSE154105], https://www.ncbi.nlm.nih.gov/geo
NV2.7513	Cole et al.^39^	repository [GSE200198 and GSE154105], https://www.ncbi.nlm.nih.gov/geo
NV2.19377	Cole et al.^39^	repository [GSE200198 and GSE154105], https://www.ncbi.nlm.nih.gov/geo
NV2.24937	Cole et al.^39^	repository [GSE200198 and GSE154105], https://www.ncbi.nlm.nih.gov/geo
NV2.6623	Cole et al.^39^	repository [GSE200198 and GSE154105], https://www.ncbi.nlm.nih.gov/geo
NV2.10352	Cole et al.^39^	repository [GSE200198 and GSE154105], https://www.ncbi.nlm.nih.gov/geo

### Experimental model and study participant details

*Nematostella vectensis* wild type and *Elav1::mOrange* were a gift from Fabian Rentzsch (University of Bergen) and maintained after.^61^ In brief, sea anemones were kept in glass Pyrex dishes filled with 14 ppt artificial sea water (ASW, Coral Pro Salt, Red Sea Aquarium System, R11220) and fed 2–3 days a week with freshly hatched brine shrimp nauplii (*Artemia* sp.). Animals that were on a spawning cycle were kept at 18 °C in darkness. The anemones were induced to spawn regularly and after fertilization and development at RT (21 °C), larvae and polyps were used at indicated ages (48–120 h postfertilization), unless stated otherwise. Sex could not be determined for the developmental stages analyzed in this study; therefore, the influence of sex on the reported results could not be assessed. No specific ethical approval is required for *Nematostella vectensis*.

### Method details

#### Behavior

##### Horizontal swimming setup

*Swimming behavior was performed with Nematostella larvae aged 48 h postfertilization (hpf) (n larvae = 123 from N spawns = 3) 72 hpf (n = 167, N = 3), 96 hpf (n = 158, N = 3) and polyps at 120 hpf (n = 187, N = 3).* In each experiment 12 larvae of a specific age were transferred into the center of a custom-made chamber (10 × 50 × 50 mm) containing 5 mL of 14 ppt ASW at RT and filmed immediately after transfer at 24 fps for 5 min using a Nikon Z50 DX 16–50 camera and a Nikkor MC 105/2.8 S lens. To illuminate the chamber evenly, four 8-RGB LED NeoPixel Sticks (Adafruit, product ID 1426) were placed at 0°, 90°, 180° and 270° at a 18 mm distance from the outer edge of the chamber (Figure S1A) and controlled through an Arduino Mega 2560 Rev3 and Arduino IDE 2.2.1 software (RGB settings: 2, 2, 2, which equals 3,0904E^13^ photons/cm^2^/s measured from the center of the arena).

##### Swimming and body shape changes in 2× microscopy

Animals in this experiment were 48 hpf (n larvae = 72, N spawns = 3), 72 hpf (*n* = 80, *N* = 3), 96 hpf (*n* = 84, *N* = 2) and 120 hpf (*n* = 80, *N* = 3), and experiments were performed at RT. The larvae were placed in a Low-Profile Open Diamond Bath Imaging Chamber (RC-26GLP, Warner Instruments) filled with 750 μL of 14 ppt ASW and placed on a Nikon Eclipse Ti2-U inverted microscope with a 2× objective lens. To simultaneously capture the variety of behaviors displayed and the larval body shape, two to three 15-s timelapse videos at 25 fps were obtained, using a Hamamatsu Orca camera (model C13440-20CU, light intensity 3.0. MS 2 with diffuser).

##### 2× swimming with MgCl_2_

Larvae aged 96 hpf were preincubated with either 40 mM MgCl_2_ in 14 ppt ASW (*n* = 84, *N* = 3), or 14 ppt ASW alone (control, *n* = 75, *N* = 3) for 5 min in a 6-well plate, then transferred into the Diamond Bath Imaging chamber and immediately filmed for 15 s as described above. However, in this dataset, due to the consideration of drug exposure time, the first video was always used for analysis.

#### Ciliary beating frequency

All experiments in this section were performed at RT in 0.2 μM filtered 14 ppt ASW.

##### Wax assay

To quantify ciliary beating frequency (CBF) larvae were placed on a glass coverslip fitted with a dental wax channel (∼250 μm wide) and allowed to adjust for 2 min in ambient light followed by 30 s under microscope light before imaging. Videos of free-moving cilia of immobilized larvae were recorded with a 40× objective (Nikon Eclipse Ti2-U inverted microscope with a Hamamatsu Orca-flash 4.0 camera (model C13440-20CU) from three body regions (aboral, side, and oral) and acquired at 150 frames per second (fps), with a 3 ms (ms) exposure and 18% light intensity, maximally 90 s from the end of the adjustment period (Figures 2A and 2B). Full body images were also taken for body metric quantification.

##### Pipette assay

To quantify CBF over longer time periods and when exposed to 40 mM MgCl_2_ in 14 ppt ASW, larvae were tethered via gentle suction using a borosilicate glass micropipette pulled by a P-97 Flaming/Brown micropipette puller (Sutter Instruments). Pipettes were broken off at the tip and fire-polished to give a rounded edge and small diameter. Perfusion of filtered ASW began immediately after tethering. Images and videos were taken for CBF analysis after 5 min (CBF 1), immediately after which the perfused liquid was either maintained or switched to the 40 mM MgCl_2_ solution and the media in the chamber carefully replaced with the experimental liquid to ensure a consistent environment. A second set of images and videos were taken after 5 min (CBF 2) (Figures 4F and 4G). Videos were acquired at 150 fps with a 2 ms exposure and 3.2% light intensity.

#### Imaging

Larvae of the desired age were selected and placed individually on a glass coverslip in 2.0 μL 0.2 μM filtered 14 ppt ASW in a light squish-prep to restrict larval movement in the z-plane. Larvae were then imaged with a 20× objective (Nikon Eclipse Ti2-U inverted microscope with a Hamamatsu Orca-flash 4.0 camera (model C13440-20CU)).

#### Immunohistochemistry

IHC was performed after.^40^^,^^62^^,^^63^ Briefly, larvae were relaxed in 2.43% MgCl_2_ in 14 ppt ASW for 15 min, fixed in 4% cold paraformaldehyde in PBS for 1 h on a rocker with ice. Larvae were then incubated in 10% DMSO in PBS 20 min at RT, and with 2% hydrogen peroxide in PBS for 15 min at RT. Larvae were washed with PBST (Triton X-0.3%), 10–15 times, and incubated in 5% Natural Goat Serum (NGS, Merck, G9023) in PBST for 1 h at RT. Incubation in primary antibody (ABs) (Mouse anti acetylated Tubulin 1:500 (Merck, T6793) and Rabbit anti-DsRed 1:100 (Takara Bio, 632496)) in 5% NGS followed at 4 °C for 60–70 h. After subsequent washes with PBST (10–15 times), and 1 h incubation with 5% NGS, secondary ABs (all 1:200: Goat anti-Mouse Cy3 (Abcam, AB97035) and Goat anti-Rabbit Alexa Fluor 647 (Thermo Fisher scientific, A-21245)) were applied overnight on a rocker at 4 °C. Samples were subsequently washed with PBST and DAPI 1:1000 in PBS (Thermo Fisher scientific, D1306) was applied for 60 min at RT. Finally, the samples were washed 3–5 times in PBS, mounted in ProLong Glass Antifade Mountant (Thermo Fisher scientific, P36980), and imaged with a Zeiss 800 Airyscan Confocal microscope Images were processed using FIJI and Adobe Photoshop.

#### Single-cell transcriptomic analysis

Whole-body single-cell RNA sequencing data of *Nematostella vectensis* was obtained from.^39^ The developmental subset of the cell atlas corresponding to stages t18h to t16d was extracted for further analysis. Transcriptional pseudobulk profiles were estimated per age group using the normalized sum of total read counts over cells of each group. Read counts were normalized to counts per million (CPM) and log-transformed in base 2. Pseudobulk expression values were used to estimate temporal expression trends of individual gene markers and the average behavior of groups of genes. The cellular landscape at age td4 (approximates t96h in Figures 5 and S5; Table S25) was analyzed using the computational framework ACTIONet.^59^ Briefly, a low-rank approximation of the normalized count matrix is obtained using the single value decomposition (SVD). This reduced data representation is subsequently decomposed using archetypal analysis to define a low-dimensional representation for each individual cell that is useful in measuring cell similarity and building a cell manifold capturing cell relationships in transcriptomic space. The network is projected in 2D coordinates for visualization using the UMAP algorithm. All these steps were implemented using the function *runACTIONet* with default parameter values. To visualize gene expression levels across the cellular landscape, a network diffusion algorithm was used over the cell network to smooth genewise sparse expression values. Network diffusion is implemented in the ACTIONet’s *networkDiffusion* function.

#### Identification of putative sensory receptor ion channel genes

To systematically identify putative voltage-gated ion channel proteins, the PFAM family HMM models Ion_trans (PF00520) and Ion_trans_2 (PF07885) were used as query for searching against the reference proteome of *Nematostella vectensis* version NV2 (wein_nvec200_tcsv2) (https://simrbase.stowers.org/starletseaanemone). The resulting protein candidates were then annotated with the best-matching human protein (Table S31). Best human protein hits were determined by querying each putative channel sequence against the complete set of human protein-coding genes using the HMMER function phmmer. Candidates matching both Ion_trans/Ion_trans_2 families and human transient receptor potential (TRP) channels were considered as putative sensory receptor genes. All sequence searches were performed using the hmmsearch program of the HMMER software package for sequence analysis v3.4 (http://hmmer.org/).^60^

### Quantification and statistical analysis

Statistical significance ∗*p* < 0.05, ∗∗*p* < 0.01, ∗∗∗*p* < 0.001.

Different letters denote significantly different mean values, where *p* < 0.05.

N indicates the number of spawns and *n* denotes the number of animals used.

Information about statistical tests, sample exclusion, and software can be found in method details; in the related section, in the Figure legends and in the supplementary material.

#### Behavior

##### Behavioral analysis (particle tracking)

Videos from the horizontal assay (Figure 1, 5 × 5 cm arena) were analyzed using Fiji ImageJ,^55^ and the plugin TrackMate^56^^,^^64^ for particle tracking. The Hessian detector and Simple LAP tracker were employed to create particle tracks. Particle track measurements (metrics) (Figures S1B–S1I) included (I) total track distance (the sum of distances between all coordinates, where d_i,i+1_ is the distance from one spot to the next spot in the track), (II) track mean speed (the mean of all momentary velocities within one track), (III) maximum speed (the momentary velocity with the highest value), (IV) minimum speed, (V) track displacement (the distance in a straight line between the first (d_i_) and last coordinate (d_x_) of one track), (VI) maximum distance (the longest distance between any two coordinates in one track), (VIII) linearity of forward progression (a relative measurement where (VII) straight line speed is divided by (II) mean speed) and (IX) confinement ratio (a relative measurement where displacement is divided by the total distance). Data were analyzed in GraphPad Prism for Windows (GraphPad software) and R Version 4.3.1 (https://www.R-project.org/).

$$\left(I\right)\;Total\;distance\;travelled=Sum_{i,j}\left(d_{i,j}\right)$$

$$\left(II\right)\;Mean\;speed=Mean\left(d_{i,i+1}\times s^{-1}\right)$$

$$\left(III\right)\;Maximum\;speed=\mathrm{Max}\left(d_{i,i+1}\times s^{-1}\right)$$

$$\left(IV\right)\;Minimum\;speed=\mathrm{Min}\left(d_{i,i+1}\times s^{-1}\right)$$

$$\left(V\right)\;Displacement=\Delta d=d_{x}-d_{i}$$

$$\left(VI\right)\;\mathrm{Max}\;distance\;travelled=Max_{i,j}\left(d_{i,j}\right)$$

(VII)Straightlinespeed=Displacement/time

(VIII)Linearityofforwardprogression=(Straightlinespeed)/(meanspeed)

(IX)Confinementratio=Displacement/(totaldistancetravelled)

The threshold for being characterized as a stationary larva in the horizontal swimming assay was displacement <0.5 mm, and maximum distance <1.0 mm (Figures 1I and 1J). The age-specific average distance the larvae swam per minute was calculated and a smoothed conditional mean line using the geom_smooth(method = “loess”) function in R was generated (Figure 1L). See also Tables S5 and S6.

##### Horizontal swimming statistical analyses

Track data (metrics) and Edge data (speed information) were extracted from TrackMate. Tracks from individuals too close to another individual, where TrackMate lost the larvae and stitching was not possible were excluded from the analysis. Data were checked for normality (D’Agostino & Pearson test, and Anderson-Darling test) and found non-normally distributed for all metrics. Therefore, a Kruskal-Wallis test with Dunn’s multiple comparisons test was used as a non-parametric alternative to ANOVA (Figures S1C–S1I). To see whether the distributions of mean speed, total distance, max distance and confinement ratio (Figures 1E–1H) differed among the ages, a Kolmogorov-Smirnov test was employed. For any correlation between metrics, we used the non-parametric Spearman’s Rank correlations. See Tables S1–S4, and S7–S13 for more information.

##### Swimming and body shape changes in 2× microscopy quantification

By using the same particle tracking software as the horizontal (5 × 5 cm) assay, swimming tracks were generated (Figure 3D). Tracks that lasted less than 1.6 s, or from larvae that were touching each other, out of focus or partially out of frame, were excluded from further analysis. The video with the lowest number of individuals meeting the exclusion criteria was retained for analysis. To measure larval body size (Area and Axis Ratio) the “Analyze particles” tool in Fiji ImageJ was used on a median-filtered (10pixels) binary version of the videos (See Videos S1 and S2). Tracks and Edge data were extracted from TrackMate and body size measurements obtained from “Analyze particles” in FIJI. Unique larval IDs and time information from these two independent datasets were matched. Subsequently, this aligned datafile was used in combination with the behavioral state analysis again, matched, using the approximate track time. In this way, each data point was assigned a momentary speed, axis ratio, and behavioral mode.

##### Behavior state analysis

To categorize behavior states (Figure 3F), tracks from the aligned datafile were resampled by a threshold of 38 μm (number of coordinates before resampling = 105 324, number of coordinates after resampling = 17 789). As some stationary larvae did not generate enough datapoints, larvae with fewer than 3 datapoints in one track were not included in the following analysis (this applied to two individuals in 48 hpf (old *n* = 72, new *n* = 70)). Displacement vectors and turning angles were calculated from successive positions. Local movement (local confinement) was measured using a 20-point rolling window, and a second rolling window averaged these values (average confinement) to capture sustained behavior. Each position was assigned to one of four behavior states:

*Pause*: Interval between consecutive samples ≥1 s.

*Swirl*: Average confinement <0.50 for ≥8 consecutive positions.

*Turn*: Turn angle ≥10°.

*Straight*: States were assigned hierarchically: pause > swirl > turn > if non applied, defined as straight. Additionally, classification errors in this mode were “smoothened” by using local confinement ≥0.90.

##### Swimming and body shape changes in 2× statistical analyses

The relative frequency (%) of each behavioral mode was calculated per individual swimming track and a Kruskal-Wallis test with Dunn’s multiple comparisons test was employed to test whether the means varied between ages (Figure 3G, see also Table S21). Correlations between any metrics and body measurements were performed by using non-parametric Spearman’s Rank correlations (Figures 3H, S3A, and S3B, for more information see Tables S22–S24). Linear models with an Age × Axis ratio interaction were used to visualize the relationship between body axis ratio and mean speed per age and per behavioral mode (Figure 3H; Table S35).

##### 2× swimming with MgCl_2_ statistical analyses

Particle tracking, body size measurements, and behavioral mode analysis were performed the same way as described above. Stationary larvae were defined as larvae with max distance <333 μm and displacement <310 μm. Differences between ratios of stationary to moving for MgCl_2_ data were calculated by a two-tailed two-proportion Z-test (Figure 4I). Differences in mean speed, max speed, and mean axis ratio between control and MgCl_2_ treated larvae were calculated by Kolmogorov-Smirnov tests and a Mann-Whitney test (Figures 4J, 4K, S4F, and S4G). For the mean speed and body axis ratio distribution data, the four quartiles (0.25, 0.50, 0.75, 1.0) were calculated in R and using these values the tracks of larvae were plotted according to which quartile they belonged to (Figures S4D and S4E). For the behavioral state analysis, one larva had fewer than 3 data points after resampling and was not included in the following analysis (this applied to MgCl_2_ (old *n* = 84, new *n* = 83)). Linear models using a Treatment × Axis ratio interaction combined with t-tests were used to evaluate differences in slopes between control and MgCl_2_ treated larvae for each behavioral mode (Figure S4I). See Tables S27–S29 and S32–S35 for more information.

#### Ciliary beating frequency

##### CBF and body metrics analysis

Analysis of recordings was conducted in FIJI ImageJ.^55^ Videos were aligned to correct for x-y drift using the plugin MultiStackReg^57^ and analyzed for ciliary beating frequency using the plugin FreQ^58^ (Figure 2D). Cilia length and tuft cilia length were measured from the point at which the cilium emerges from the larval body to its tip. Each point represents the average of one individual larva, comprising an average of 8–12 cilia. Length and width of larvae oriented in the horizontal plane were measured, and body axis ratio calculated by dividing length by width. Due to unequal variance between age groups, differences in body axis ratio were tested by Brown-Forsythe ANOVA test and Dunnett’s T3 multiple comparisons test (Figures 2E and 2F; Table S20). Data were analyzed in GraphPad Prism for Windows (GraphPad software, www.graphpad.com) and R Version 4.5.0 (https://www.R-project.org/).

##### Wax assay statistical analyses

All data were checked for normality using both the D’Agostino & Pearson test and the Anderson-Darling test. To test for differences in both cilia and body metrics over development, the following analyses were performed: Differences in CBF (48–96 hpf *n* = 10–18, *N* = 6) were assessed using a Kolmogorov-Smirnov test. Hartigan’s Dip Test was applied to assess multimodality (Figure 2D). A small number of groups within the ciliary length data were determined to be non-normal. However, due to sample size and differing variabilities, an ANOVA was determined to be the best method for statistical analysis. Therefore, for cilia length data (48–96 hpf *n* = 21–30, *N* = 5; 120 hpf *n* = 28–31, *N* = 1) within region-groups, a one-way ANOVA with Šídák’s multiple comparisons was performed; testing for differences in cilia length within age-group was also done using a mixed-effects analysis with Šídák’s multiple comparisons (results not described here) (Figure 2E). For body axis ratio (48–96 hpf *n* = 31–37, *N* = 4; 120 hpf: *n* = 31, *N* = 1), a one-way ANOVA with Dunnett’s T3 multiple comparisons was performed (Figure 3B), and, tuft (cilia) length (48–96 hpf *n* = 16–21, *N* = 5; 120 hpf *n* = 13, *N* = 1) was compared using a Kruskal-Wallis test with Dunn’s multiple comparisons (Figure 2F). For more information see Tables S15–S19.

##### Pipette assay statistical analyses

CBF data in pipette-attached larvae were determined to be non-parametric. To compare the control (CBF 1) and treatment (CBF 2) conditions for each treatment group, a Wilcoxon matched-pairs signed rank test was done (ASW *n* = 8, *N* = 2; MgCl2 *n* = 10, *N* = 2) (Figure 4H). See also Table S26.

#### Immunohistochemistry

##### Elav::mOrange neuronal quantification

*Elav::mOrange* positive neurons visualized through immunohistochemistry were counted manually for 48 hpf (*n* = 3), 72 hpf(*n* = 5), 96 hpf (*n* = 5) and 120 hpf (*n* = 6). Two independent counts were averaged and data plotted as mean ± SEM (Figure S4A). For more information see Table S30.

## Supplemental information

Document S1. Figures S1–S4 and Tables S1–S35 related to Figures 1–4 and S1–S4
