# Supplementary material for: Developmentally timed sensory integration enables efficient larval dispersal
Source: iScience. 2026 Aug 13;29(9):117222. doi: 10.1016/j.isci.2026.117222 (PMC13499137; doi:10.1016/j.isci.2026.117222)
Supplement: Supplementary file 1 [file mmc1.pdf]

iScience, Volume *29*

Supplemental information

Developmentally timed sensory integration enables efficient larval dispersal

Marte Lønnum, Madeline M. Schuldt, Johan Hovland, Jose Davila-Velderrain, and Lena van Giesen

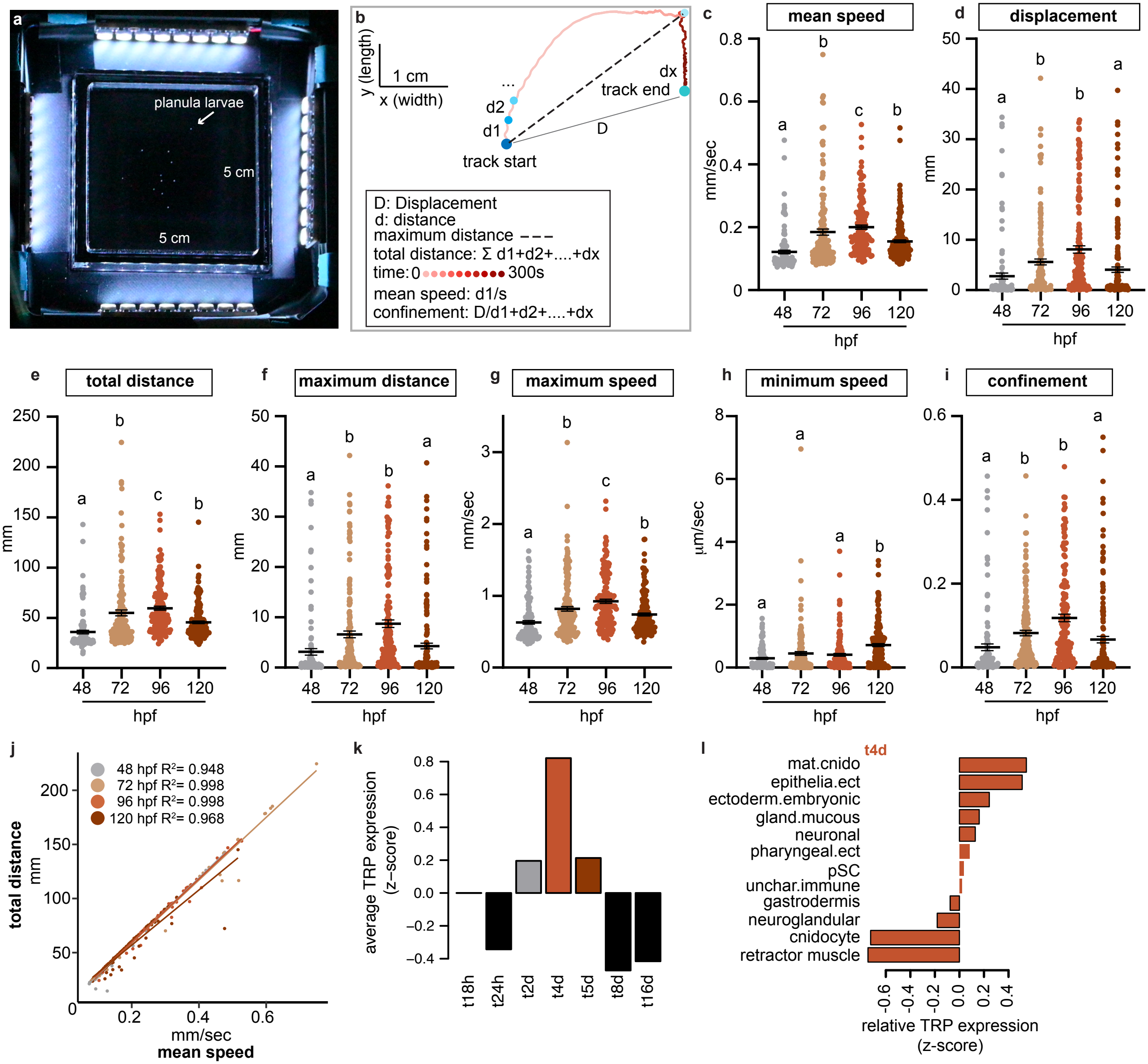

a
planula larvae
5 cm
5 cm
b
y (length)
1 cm
x (width)
d2
d1
track start
dx
track end
D
D: Displacement
d: distance
maximum distance
total distance: Σ d1+d2+....+dx
time: 0 300s
mean speed: d1/s
confinement: D/d1+d2+....+dx
c
mean speed
mm/sec
hpf
d
displacement
mm
e
total distance
f
maximum distance
g
maximum speed
h
minimum speed
μm/sec
i
confinement
j
48 hpf R²= 0.948
72 hpf R²= 0.998
96 hpf R²= 0.998
120 hpf R²= 0.968
total distance
mean speed
k
average TRP expression (z-score)
t18h
t24h
t2d
t4d
t5d
t8d
t16d
l
t4d
mat.cnido
epithelia.ect
ectoderm.embryonic
gland.mucous
neuronal
pharyngeal.ect
pSC
unchar.immune
gastrodermis
neuroglandular
cnidocyte
retractor muscle
relative TRP expression (z-score)

Figure S1

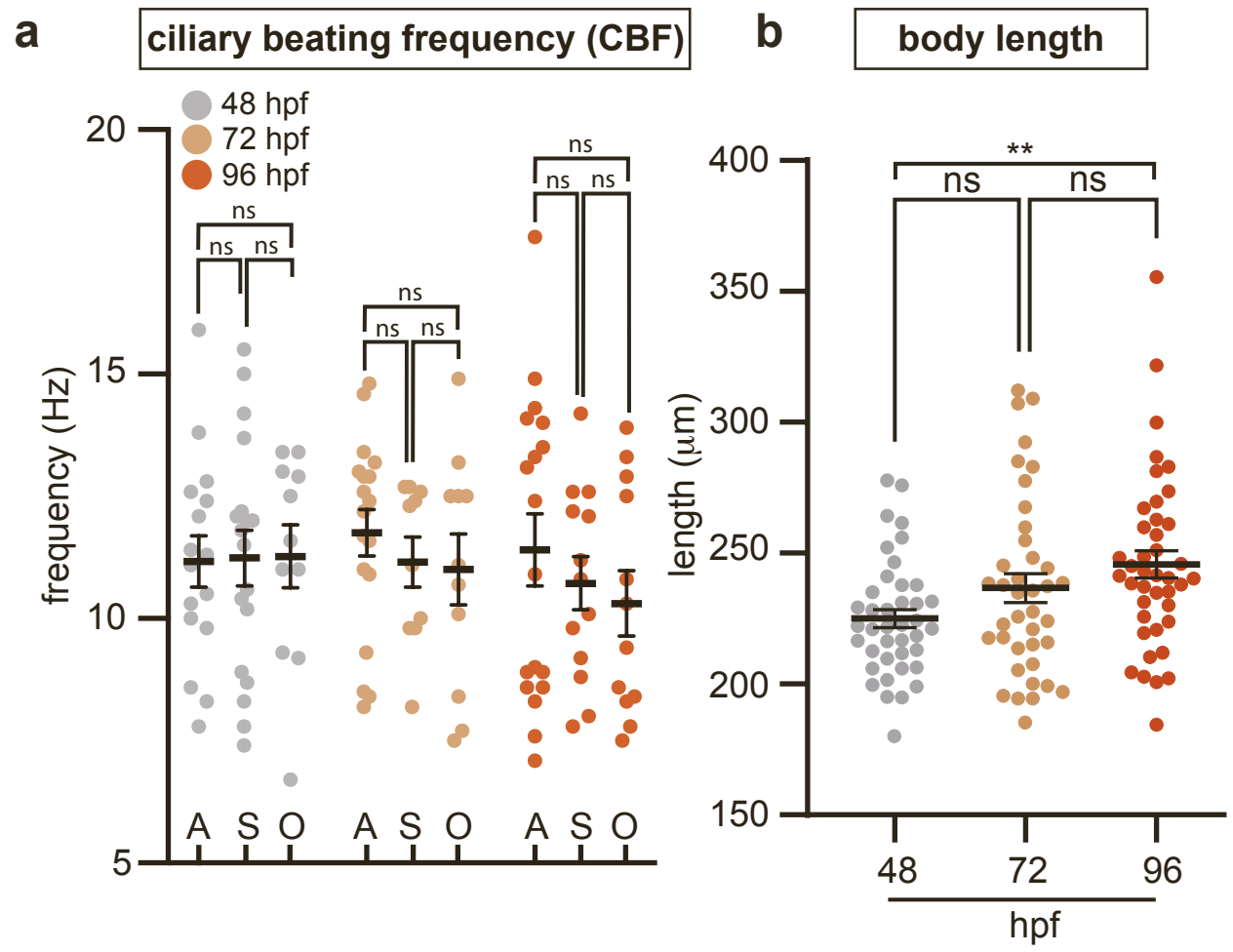
a
ciliary beating frequency (CBF)
48 hpf
72 hpf
96 hpf
frequency (Hz)
ns
A S O A S O A S O
b
body length
length (µm)
**
ns
48 72 96
hpf

Figure S2

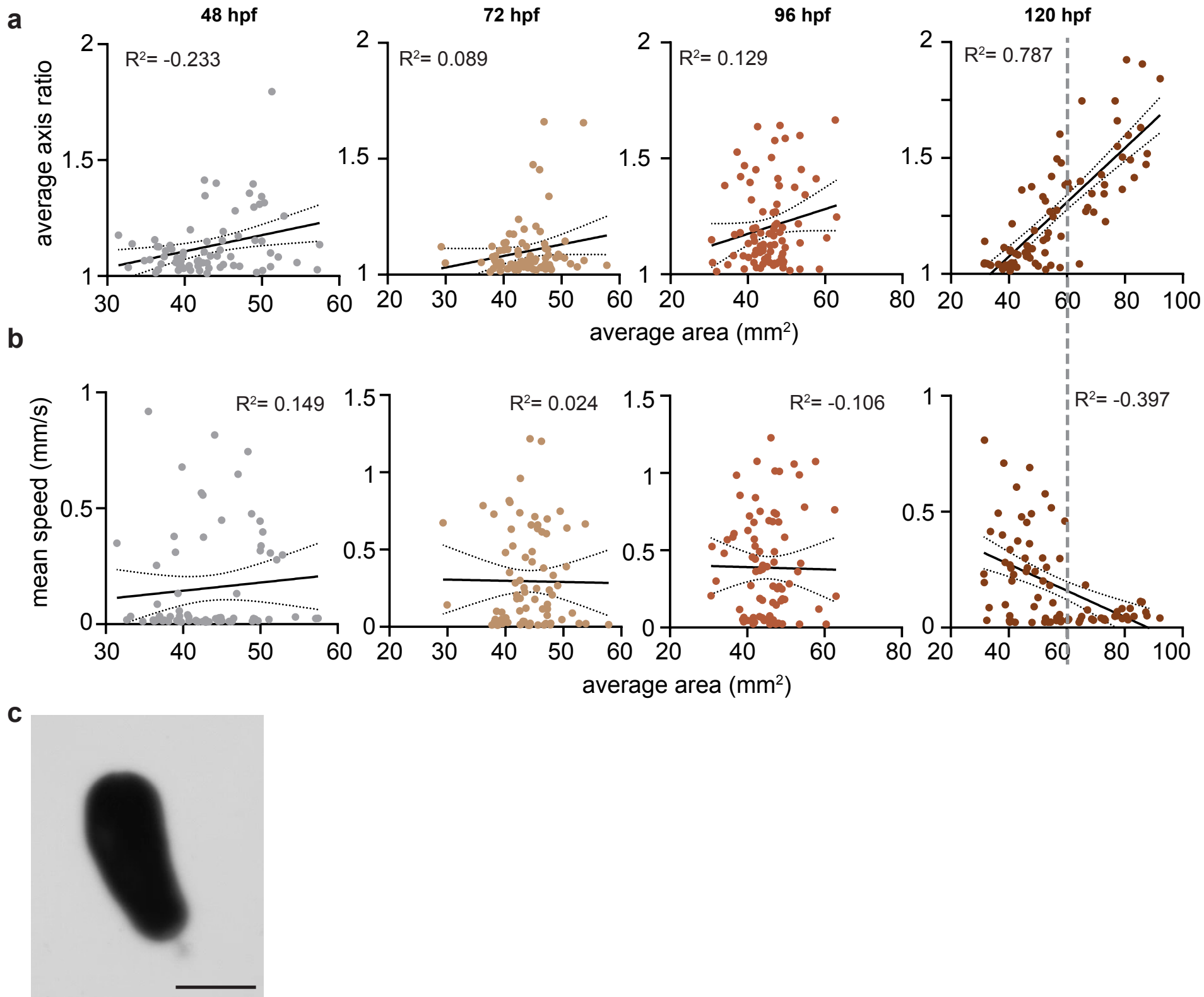

a
48 hpf
72 hpf
96 hpf
120 hpf
average axis ratio
R²= -0.233
R²= 0.089
R²= 0.129
R²= 0.787
average area (mm²)
b
mean speed (mm/s)
R²= 0.149
R²= 0.024
R²= -0.106
R²= -0.397
average area (mm²)
c

Figure S3

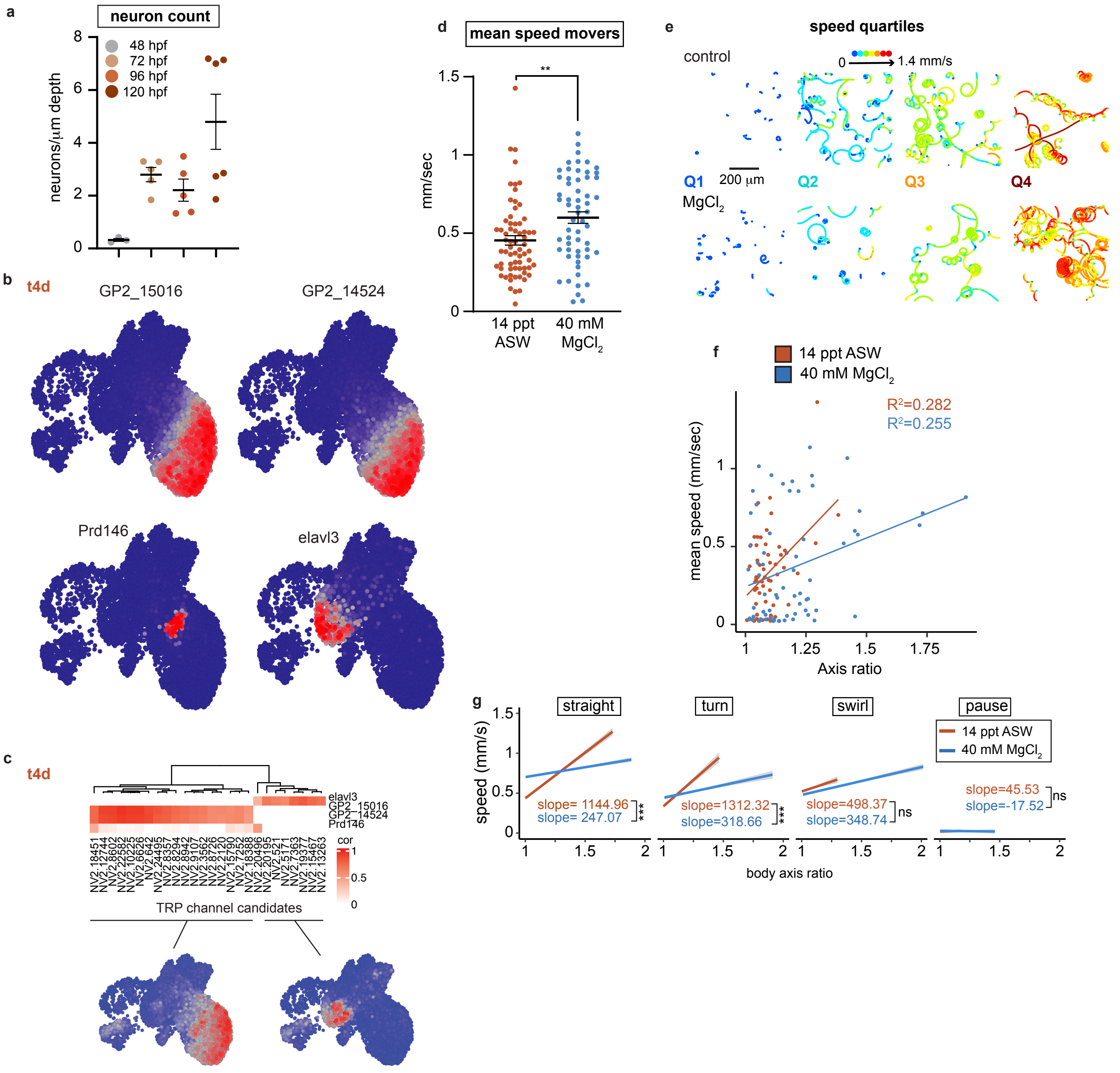

a
neuron count
neurons/µm depth
48 hpf
72 hpf
96 hpf
120 hpf
b
t4d
GP2_15016
GP2_14524
Prd146
elavl3
c
t4d
elavl3
GP2_15016
GP2_14524
Prd146
cor
TRP channel candidates
d
mean speed movers
mm/sec
14 ppt ASW
40 mM MgCl2
e
speed quartiles
control
0
1.4 mm/s
200 µm
Q1
Q2
Q3
Q4
MgCl2
f
14 ppt ASW
40 mM MgCl2
R2=0.282
R2=0.255
mean speed (mm/sec)
Axis ratio
g
speed (mm/s)
straight
turn
swirl
pause
14 ppt ASW
40 mM MgCl2
slope= 1144.96
slope= 247.07
slope=1312.32
slope=318.66
slope=498.37
slope=348.74
ns
slope=45.53
slope=-17.52
ns
body axis ratio

Figure S4

Supplemental figure legends

Figure S1: Tracking set up with metrics and expression of TRP channels. a) The behavioral arena used for horizontal swimming experiments. **b**) Explanations of various metrics calculated by the tracking software. **c**) Mean speed. **d**) Displacement. **e**) Total distance. **f**) Maximum distance. **g**) Maximum speed. **h**) Minimum speed. **i**) Confinement ratio. **c-i**) Each point represents one individual larva 48hpf (n=123, N=3), 72hpf (n=167, N=3), 96hpf (n=158, N=3) and 120hpf (n=187, N=3); lines represent the mean value ± SEM; letters denote statistical significance by Kruskal-Wallis test and Dunn's multiple comparisons. **c-i)** For more information see also **Supplementary tables S7-14**. **j**) Correlation between mean speed and total distance for each age. **k**) Average relative expression profile of putative TRP channels across developmental time. Data shows the average value of relative expression (z-scaled) profiles of putative TRP channel encoding genes (n=50 genes). **l**) Average relative expression profile of putative TRP channels across cell groups at developmental time td4. Data shows similar values as those in k) but estimated by cell group at the time with highest expression (td4).

Figure S2: Individual data points for ciliary beating frequency (CBF) and body length across ages. a) Ciliary beating at frequencies ranging from 7 to 18 Hz with no significant differences between the means (48-96hpf n=10-18, N=6). Lines at mean ± SEM; letters denote statistical significance of ns p>0.05 by mixed-effects analysis with Šídák's multiple comparisons. **b)** Body length of larvae shows an increasing trend with age (48-96hpf n=38-40, N=5); stars denote statistical significance of **p<0.01 by Dunnett's T3 multiple comparisons. Lines at means ± SEM. See also **Supplementary table S18-19**.

Figure S3: Body shape measurements and correlations. a) Linear regression lines with 95% confidence interval, and Spearman's Rank correlation coefficient between the average body area and average axis ratio for each age (48hpf: 3N, 72n, 72hpf: 3N, 80n, 96hpf: 2N, 84n, 120hpf: 3N, 80n). **b)** Linear regression lines with 95% confidence interval, and Spearman's Rank correlation coefficient between the average body area and mean speed for each age (48hpf: n=72, 3N, 72hpf: n=80, N=3, 96hpf: n=84, N=2, 120hpf: n=80, N=3). **c)** Representative still-image of a 96hpf larvae actively swimming with a visible tuft, taken from 2x assay video, scale bar: 200 μm. See also **Supplementary table S23-24.**

Figure S4: Spatial expression pattern of markers and $MgCl_2$ treatment data. a) Quantification of neuron count (neuron number per μm depth in *Elav::mOrange* positive animals) per age**. b)** 2D projection of single-cell expression values (right) of marker genes across cell landscape at developmental time t4d. **c)** Correlation between marker expression values (rows) and TRP channel candidates (columns) preferentially expressed in epithelial cells (GP2+) or neuronal cells (elavl3+). 2D cell landscape inserts show projections of average expression values of the corresponding TRP channel candidates. **d)** Tracks of larvae sorted into the defined speed quartiles (Q1-Q4) from the control and $MgCl_2$ treated larvae (Ctrl n=84, $MgCl_2$ n=88). Colors denote momentary speed. **e)** Tracks of larvae sorted into the defined axis ratio quartiles (Q1-Q4)

from the control and $MgCl_2$treated larvae (Ctrl n=84, $MgCl_2$ n=88). Colors denote momentary speed. f**)** Distributions of mean speeds between the control and $MgCl_2$ treated larvae. Line at the mean, ns p=0.0575981 by Kolmogorov-SmirnovMann-Whitney test. **g)** Mean speed for moving animals. **p<0.0015 by Mann-Whitney test (Ctrl n=67, $MgCl_2$ n=58). **h)** Spearman's Rank correlation between axis ratio and mean speed for the two treatments. **i)** Linear regression models with 95% confidence intervals show the relationship between momentary body axis ratio and momentary swimming speed per behavioral mode for the two treatments ($MgCl_2$ n=83, Ctrl n=75). Significance of slope differences annotated as ns p>0.05 and ***p<0.001 by t-tests on the interaction terms (Treatment*Axis ratio). For more information see **Supplementary table S30-37**.

Table S1: Descriptive statistics and Kolmogorov-Smirnov test related to Figure 1E.

Descriptive statistics: Mean speed (μm/sec)				
Age	48hpf	72hpf	96hpf	120hpf
Number of values	123	167	158	187
Mean	121.6	184.8	200.3	155.2
Std. Deviation	63.82	126.5	86.05	61.44
Std. Error of Mean	5.755	9.791	6.846	4.493
Kolmogorov-Smirnov test on Mean speed				
Comparison	D-statistic	p-value		
48hpf vs 72hpf	0.4188	3.451e-11		
48hpf vs 96hpf	0.59344	2.2e-16		
48hpf vs 120hpf	0.53254	2.2e-16		
72hpf vs 96hpf	0.26125	3.178e-05		
72hpf vs 120hpf	0.15141	0.03548		
96hpf vs 120hpf	0.3126	1.077e-07		

Table S2: Descriptive statistics and Kolmogorov-Smirnov test related to Figure 1F.

Descriptive statistics: Total distance (μm)				
Age	48hpf	72hpf	96hpf	120hpf
Number of values	123	166	158	187
Mean	35878	54892	59549	45489
Std. Deviation	19207	36898	25349	16532
Std. Error of Mean	1732	2864	2017	1209
Kolmogorov-Smirnov test on Total distance				
Comparison	D-statistic	p-value		
48hpf vs 72hpf	0.41037	9.259e-11		
48hpf vs 96hpf	0.61243	2.2e-16		
48hpf vs 120hpf	0.54067	2.2e-16		
72hpf vs 96hpf	0.26727	1.898e-05		
72hpf vs 120hpf	0.14471	0.0503		
96hpf vs 120hpf	0.31598	7.481e-08		

Table S3: Descriptive statistics and Kolmogorov-Smirnov test related to Figure 1G.

Descriptive statistics: Max distance (μm)				
Age	48hpf	72hpf	96hpf	120hpf
Number of values	123	167	158	187
Mean	3134	6594	8744	4289
Std. Deviation	7097	8744	9444	7682
Std. Error of Mean	639.9	676.6	751.3	561.8

Kolmogorov-Smirnov test on Max distance				
Comparison	D-statistic	p-value		
48hpf vs 72hpf	0.40704	1.36e-10		
48hpf vs 96hpf	0.54456	2.2e-16		
48hpf vs 120hpf	0.12808	0.1752		
72hpf vs 96hpf	0.17066	0.01792		
72hpf vs 120hpf	0.34266	2.15e-09		
96hpf vs 120hpf	0.44016	7.772e-15		

Table S4: Descriptive statistics and Kolmogorov-Smirnov test related to Figure 1H.

Descriptive statistics: Confinement ratio				
Age	48hpf	72hpf	96hpf	120hpf
Number of values	123	167	158	187
Mean	0.04833	0.08232	0.1182	0.06658
Std. Deviation	0.09058	0.08399	0.1127	0.1069
Std. Error of Mean	0.008167	0.006500	0.008968	0.007819
Kolmogorov-Smirnov test on confinement ratio				
Comparison	D-statistic	p-value		
48hpf vs 72hpf	0.4067	1.415e-10		
48hpf vs 96hpf	0.51019	4.441e-16		
48hpf vs 120hpf	0.10647	0.3695		
72hpf vs 96hpf	0.14633	0.06243		
72hpf vs 120hpf	0.32717	1.334e-08		
96hpf vs 120hpf	0.41539	2.924e-13		

Table S5: Quantification of moving vs stationary larvae related to Figure 1J.

Quantification of moving vs. stationary larvae					
Age	# stationary	# moving	# Total	% stationary	% moving
48hpf	69	54	123	56	44
72hpf	33	134	167	20	80
96hpf	17	140	157	11	89
120hpf	89	98	187	48	52

Table S6: Calculation of distance moved per minute related to Figure 1L.

Distance moved per minute (mm)				
Time (min)	48hpf	72hpf	96hpf	120hpf
1	7.03 mm ± 0.41 SEM	14.33 mm± 0.87 SEM	17.43 mm ± 0.91 SEM	10.65 mm ±0.51 SEM
2	7.34 mm ± 0.49 SEM	11.33 mm ± 0.69 SEM	12.20 mm ± 0.59 SEM	8.88 mm± 0.28 SEM
3	7.34 mm± 0.44 SEM	10.25 mm ± 0.63 SEM	9.92 mm ± 0.42 SEM	8.75 mm ± 0.26 SEM
4	7.30 mm ± 0.43 SEM	9.65 mm ± 0.54 SEM	10.10 mm ± 0.42 SEM	8.59 mm ± 0.22 SEM
5	6.77 mm ± 0.33 SEM	9.32 mm ± 0.48 SEM	9.91 mm ± 0.42 SEM	8.73 mm± 0.28 SEM

Table S7: Kruskal Wallis test and Dunn's multiple comparisons related to Figure S1C.

Kruskal Wallis results				
P value	<0.0001			
Exact or approximate P value?	Approximate			
P value summary	***			
Do the medians vary signif. (P < 0.05)?	Yes			
Number of groups	4			
Kruskal-Wallis statistic	122.4			
Multiple comparisons				
Dunn's multiple comparisons test	Mean rank diff.	Significant?	Summary	Adjusted P Value
48hpf vs. 72hpf	-151.6	Yes	***	<0.0001
48hpf vs. 96hpf	-243.0	Yes	***	<0.0001
48hpf vs. 120hpf	-151.7	Yes	***	<0.0001
72hpf vs. 96hpf	-91.40	Yes	***	<0.0001
72hpf vs. 120hpf	-0.1417	No	ns	>0.9999
96hpf vs. 120hpf	91.26	Yes	***	<0.0001
Compact letter display				
48hpf	A			
72hpf	B			
96hpf	C			
120hpf	B			

Table S8: Descriptive statistics. Kruskal Wallis test and Dunn's multiple comparisons related to Figure S1D.

Descriptive statistics: Displacement (µm)				
	48hpf	72hpf	96hpf	120hpf
Number of values	123	166	158	187
Mean	2791	5607	8088	4071
Std. Deviation	6798	7743	9107	7531
Std. Error of Mean	613.0	601.0	724.5	550.7
Kruskal Wallis results				
P value	<0.0001			
Exact or approximate P value?	Approximate			

P value summary	***			
Do the medians vary signif. (P < 0.05)?	Yes			
Number of groups	4			
Kruskal-Wallis statistic	103.0			
Multiple comparisons				
Dunn's multiple comparisons test	Mean rank diff.	Significant?	Summary	Adjusted P Value
48hpf vs. 72hpf	-145.9	Yes	***	<0.0001
48hpf vs. 96hpf	-194.9	Yes	***	<0.0001
48hpf vs. 120hpf	-50.69	No	ns	0.1028
72hpf vs. 96hpf	-49.05	No	ns	0.0959
72hpf vs. 120hpf	95.17	Yes	***	<0.0001
96hpf vs. 120hpf	144.2	Yes	***	<0.0001
Compact letter display				
48hpf	A			
72hpf	B			
96hpf	B			
120hpf	A			

Table S9: Kruskal Wallis test and Dunn's multiple comparisons related to Figure S1E.

Descriptive statistics: Total distance (µm)				
Age	48hpf	72hpf	96hpf	120hpf
Number of values	123	166	158	187
Mean	35878	54892	59549	45489
Std. Deviation	19207	36898	25349	16532
Std. Error of Mean	1732	2864	2017	1209
Kruskal Wallis results				
P value	<0.0001			
Exact or approximate P value?	Approximate			
P value summary	***			
Do the medians vary signif. (P < 0.05)?	Yes			
Number of groups	4			
Kruskal-Wallis statistic	125.5			
Multiple comparisons				
Dunn's multiple comparisons test	Mean rank diff.	Significant?	Summary	Adjusted P Value
48hpf vs. 72hpf	-156.6	Yes	***	<0.0001
48hpf vs. 96hpf	-245.6	Yes	***	<0.0001
48hpf vs. 120hpf	-150.5	Yes	***	<0.0001
72hpf vs. 96hpf	-89.02	Yes	***	<0.0001
72hpf vs. 120hpf	6.065	No	ns	>0.9999
96hpf vs. 120hpf	95.08	Yes	***	<0.0001
Compact letter display				
48hpf	A			

72hpf	B			
96hpf	C			
120hpf	B			

Table S10: Kruskal Wallis test and Dunn’s multiple comparisons related to Figure S1F.

Descriptive statistics: Max distance (µm)				
Age	48hpf	72hpf	96hpf	120hpf
Number of values	123	167	158	187
Mean	3134	6594	8744	4289
Std. Deviation	7097	8744	9444	7682
Std. Error of Mean	639.9	676.6	751.3	561.8
Kruskal Wallis results				
P value	<0.0001			
Exact or approximate P value?	Approximate			
P value summary	***			
Do the medians vary signif. (P < 0.05)?	Yes			
Number of groups	4			
Kruskal-Wallis statistic	109.0			
Multiple comparisons				
Dunn's multiple comparisons test	Mean rank diff.	Significant?	Summary	Adjusted P Value
48hpf vs. 72hpf	-148.7	Yes	***	<0.0001
48hpf vs. 96hpf	-197.9	Yes	***	<0.0001
48hpf vs. 120hpf	-46.84	No	ns	0.1672
72hpf vs. 96hpf	-49.26	No	ns	0.0932
72hpf vs. 120hpf	101.8	Yes	***	<0.0001
96hpf vs. 120hpf	151.1	Yes	***	<0.0001
Compact letter display				
48hpf	A			
72hpf	B			
96hpf	B			
120hpf	A			

Table S11: Descriptive statistics. Kruskal Wallis test and Dunn’s multiple comparisons related to Figure S1G.

Descriptive statistics: Maximum speed (µm/sec)				
Age	48hpf	72hpf	96hpf	120hpf
Number of values	123	167	158	187
Mean	629.6	817.2	923.1	739.8
Std. Deviation	282.2	408.7	375.2	281.7
Std. Error of Mean	25.45	31.62	29.85	20.60
Kruskal Wallis results				

P value	<0.0001			
Exact or approximate P value?	Approximate			
P value summary	***			
Do the medians vary signif. (P < 0.05)?	Yes			
Number of groups	4			
Kruskal-Wallis statistic	70.36			
Multiple comparisons				
Dunn's multiple comparisons test	Mean rank diff.	Significant?	Summary	Adjusted P Value
48hpf vs. 72hpf	-112.7	Yes	***	<0.0001
48hpf vs. 96hpf	-183.9	Yes	***	<0.0001
48hpf vs. 120hpf	-94.38	Yes	***	<0.0001
72hpf vs. 96hpf	-71.16	Yes	**	0.0028
72hpf vs. 120hpf	18.32	No	ns	>0.9999
96hpf vs. 120hpf	89.49	Yes	***	<0.0001
Compact letter display				
48hpf	A			
72hpf	B			
96hpf	C			
120hpf	B			

Table S12: Descriptive statistics. Kruskal Wallis test and Dunn's multiple comparisons related to Figure S1H.

Descriptive statistics: Minimum speed (µm/sec)				
Age	48hpf	72hpf	96hpf	120hpf
Number of values	123	166	158	187
Mean	0.2935	0.4501	0.4068	0.7170
Std. Deviation	0.3403	0.7338	0.5443	0.6651
Std. Error of Mean	0.03069	0.05695	0.04330	0.04864
Kruskal Wallis results				
P value	<0.0001			
Exact or approximate P value?	Approximate			
P value summary	***			
Do the medians vary signif. (P < 0.05)?	Yes			
Number of groups	4			
Kruskal-Wallis statistic	46.74			
Multiple comparisons				
Dunn's multiple comparisons test	Mean rank diff.	Significant?	Summary	Adjusted P Value
48hpf vs. 72hpf	-35.09	No	ns	0.6443
48hpf vs. 96hpf	-36.78	No	ns	0.5698
48hpf vs. 120hpf	-131.0	Yes	***	<0.0001
72hpf vs. 96hpf	-1.691	No	ns	>0.9999
72hpf vs. 120hpf	-95.92	Yes	***	<0.0001
96hpf vs. 120hpf	-94.23	Yes	***	<0.0001
Compact letter display				

48hpf	A			
72hpf	A			
96hpf	A			
120hpf	B			

Table S13: Kruskal Wallis test and Dunn’s multiple comparisons related to Figure S1I.

Descriptive statistics: Confinement ratio				
Age	48hpf	72hpf	96hpf	120hpf
Number of values	123	167	158	187
Mean	0.04833	0.08232	0.1182	0.06658
Std. Deviation	0.09058	0.08399	0.1127	0.1069
Std. Error of Mean	0.008167	0.006500	0.008968	0.007819
Kruskal Wallis results				
P value	<0.0001			
Exact or approximate P value?	Approximate			
P value summary	***			
Do the medians vary signif. (P < 0.05)?	Yes			
Number of groups	4			
Kruskal-Wallis statistic	88.31			
Multiple comparisons				
Dunn's multiple comparisons test	Mean rank diff.	Significant?	Summary	Adjusted P Value
48hpf vs. 72hpf	-129.5	Yes	***	<0.0001
48hpf vs. 96hpf	-172.1	Yes	***	<0.0001
48hpf vs. 120hpf	-30.47	No	ns	0.9148
72hpf vs. 96hpf	-42.58	No	ns	0.2190
72hpf vs. 120hpf	99.00	Yes	***	<0.0001
96hpf vs. 120hpf	141.6	Yes	***	<0.0001
Compact letter display				
48hpf	A			
72hpf	B			
96hpf	B			
120hpf	A			

Table S14: Spearman Correlation of Total distance vs Mean speed related to Figure S1J.

Spearman Correlation: Total distance vs. Mean speed		
Age	Correlation coefficient R^2	n
48hpf	0.948	123
72hpf	0.998	167
96hpf	0.998	157
120hpf	0.968	187

Table S15: Kolmogorov-Smirnov test and Hartigan's Dip test related to Figure 2D.

KS test results (within region-group)				
Group 1	Group 2	D-statistic	p-value	
48 aboral	72 aboral	0.29167	0.3614	
48 aboral	96 aboral	0.31944	0.2656	
72 aboral	96 aboral	0.27778	0.458	
48 oral	72 oral	0.27273	0.6414	
48 oral	96 oral	0.46667	0.1227	
72 oral	96 oral	0.22727	0.8221	
48 side	72 side	0.27778	0.6101	
48 side	96 side	0.18376	0.8776	
72 side	96 side	0.26923	0.6551	
Hartigan's Dip test: Ciliary beating frequency				
Age	Region	n	Hartigan's Dip Test P value	Hartigan's Dip Test Dip statistic
48 hpf	Aboral	16	0.8995	0.0625
48 hpf	Side	11	0.8003	0.1000
48 hpf	Oral	18	0.4496	0.0645
72 hpf	Aboral	17	0.6496	0.0710
72 hpf	Side	11	0.0807	0.1340
72 hpf	Oral	10	0.6311	0.0892
96 hpf	Aboral	18	0.0238	0.1180
96 hpf	Side	12	0.9304	0.0662
96 hpf	Oral	13	0.4900	0.0914

Table S16: Descriptive statistics. ANOVA and Šídák's multiple comparisons test related to Figure 2E. Additional Mixed-effects analysis and Šídák's multiple comparisons

Descriptive statistics: Ciliary length (µm)												
	Aboral 48 hpf	Aboral 72 hpf	Aboral 96 hpf	Aboral 120 hpf	Side 48 hpf	Side 72 hpf	Side 96 hpf	Side 120 hpf	Oral 48 hpf	Oral 72 hpf	Oral 96 hpf	Oral 120 hpf
Number of values	25	27	28	26	30	30	27	30	21	28	27	31
Mean	10.45	10.89	11.40	11.58	15.55	16.63	16.31	13.94	9.894	11.42	11.49	11.93
Std. Deviation	1.506	1.266	1.450	1.432	1.296	0.9047	1.421	2.382	1.960	1.542	1.265	2.057
Std. Error of Mean	0.3012	0.2436	0.2739	0.2808	0.2366	0.1652	0.2734	0.4349	0.4276	0.2913	0.2434	0.3695
ANOVA Results (within region-group)												
F	60.03											
P value	<0.0001											
P value summary	***											
Significant diff. among means (P < 0.05)?	Yes											
R squared	0.6750											
Multiple comparisons (within region-group)												
Šídák's multiple comparisons test	Mean Diff.	95.00% CI of diff.	Below threshold?	Summary	Adjusted P Value							
Aboral 48 vs. Aboral 72	-0.4422	-1.769 to 0.8843	No	ns	0.9990							
Aboral 48 vs. Aboral 96	-0.9514	-2.266 to 0.3636	No	ns	0.4253							
Apical 48 vs. Aboral 120	-1.137	-2.475 to 0.2022	No	ns	0.1826							
Aboral 72 vs. Aboral 96	-0.5092	-1.798 to 0.7798	No	ns	0.9921							
Aboral 72 vs. Aboral 120	-0.6943	-2.007 to 0.6188	No	ns	0.8840							
Aboral 96 vs. Aboral 120	-0.1851	-1.487 to 1.116	No	ns	>0.9999							
Side 48 vs. Side 72	-1.076	-2.310 to 0.1582	No	ns	0.1528							

Side 48 vs. Side 96	-0.7577	-2.025 to 0.5101	No	ns	0.7456							
Side 48 vs. Side 120	1.606	0.3717 to 2.840	Yes	**	0.0020							
Side 72 vs. Side 96	0.3181	-0.9497 to 1.586	No	ns	>0.9999							
Side 72 vs. Side 120	2.681	1.447 to 3.915	Yes	***	<0.0001							
Side 96 vs. Side 120	2.363	1.096 to 3.631	Yes	***	<0.0001							
Oral 48 vs. Oral 72	-1.527	-2.907 to -0.1474	Yes	*	0.0174							
Oral 48 vs. Oral 96	-1.597	-2.987 to -0.2064	Yes	*	0.0112							
Oral 48 vs. Oral 120	-2.035	-3.385 to -0.6839	Yes	***	0.0002							
Oral 72 vs. Oral 96	-0.06996	-1.359 to 1.219	No	ns	>0.9999							
Oral 72 vs. Oral 120	-0.5076	-1.754 to 0.7383	No	ns	0.9889							
Oral 96 vs. Oral 120	-0.4377	-1.696 to 0.8204	No	ns	0.9982							
Mixed-effects Results (within age-groups)												
Fixed effect (type III)	P value	P value summary	Statistically significant (P < 0.05)?	F (DFn. DFd)	Geisser-Greenhouse's epsilon							
Treatment (between columns)	<0.0001	***	Yes	F (6.543. 171.3) = 63.02	0.5948							
Random effects	SD	Variance										
Individual (between rows)	0.3608	0.1301										
Residual	1.548	2.395										
Was the matching effective?												
Chi-square. df	3.373. 1											
P value	0.0663											

P value summary	ns											
Is there significant matching (P < 0.05)?	No											
Multiple comparisons (within age-groups)												
Šídák's multiple comparisons test	Mean Diff.	95.00% CI of diff.	Below threshold?	Summary	Adjusted P Value							
48 - Aboral vs. 48 - Side	-5.082	-5.916 to -4.247	Yes	***	<0.0001							
48 - Aboral vs. 48 - Oral	0.3376	-1.266 to 1.941	No	ns	0.9998							
48 - Side vs. 48 - Oral	5.462	3.852 to 7.072	Yes	***	<0.0001							
72 - Aboral vs. 72 - Side	-5.805	-6.757 to -4.853	Yes	***	<0.0001							
72 - Aboral vs. 72 - Oral	-0.4381	-1.357 to 0.4811	No	ns	0.8528							
72 - Side vs. 72 - Oral	5.249	4.093 to 6.406	Yes	***	<0.0001							
96 - Aboral vs. 96 - Side	-4.884	-6.072 to -3.696	Yes	***	<0.0001							
96 - Aboral vs. 96 - Oral	-0.06722	-1.095 to 0.9610	No	ns	>0.9999							
96 - Side vs. 96 - Oral	4.817	3.580 to 6.053	Yes	***	<0.0001							
120 - Aboral vs. 120 - Side	-2.469	-4.334 to -0.6033	Yes	**	0.0039							
120 - Aboral vs. 120 - Oral	-0.6572	-2.208 to 0.8937	No	ns	0.9258							
120 - Side vs. 120 - Oral	2.009	0.3645 to 3.654	Yes	**	0.0085							

Table S17: Descriptive statistics. Kruskal Wallis test and Dunn's multiple comparisons test related to Figure 2F.

Descriptive statistics: Tuft cilia length (µm)				
Age	48 hpf	72 hpf	96 hpf	120 hpf
Number of values	16	21	20	13
Mean	36.81	67.91	96.07	78.86
Std. Deviation	13.30	18.82	28.41	48.87
Std. Error of Mean	3.324	4.106	6.353	13.55
Kruskal Wallis results				
P value	<0.0001			
Exact or approximate P value?	Approximate			
P value summary	***			
Do the medians vary signif. (P<0.05)?	Yes			
Number of groups	4			
Kruskal-Wallis statistic	31.48			
Multiple comparisons				
Dunn's multiple comparisons test	Mean rank diff.	Significant?	Summary	Adjusted P Value
48 vs. 72	-23.34	Yes	**	0.0033
48 vs. 96	-38.16	Yes	***	<0.0001
48 vs. 120	-23.74	Yes	*	0.0107
72 vs. 96	-14.83	No	ns	0.1183
72 vs. 120	-0.3993	No	ns	>0.9999
96 vs. 120	14.43	No	ns	0.2797
Compact letter display				
48	A			
72	B			
96	B			
120	B			

Table S18: Descriptive statistics. Mixed effect analysis and Šídák's multiple comparisons test related to Figure S2A.

Descriptive statistics: CBF (Hz)									
	48hpf – Aboral	48hpf - Side	48hpf - Oral	72hpf - Aboral	72hpf - Side	72hpf - Oral	96hpf - Aboral	96hpf - Side	96hpf - Oral
Number of values	16	18	11	17	10	11	18	13	12
Mean	11.21	11.24	11.26	11.76	11.16	11.01	11.43	10.81	10.31
Std. Deviation	2.120	2.421	2.096	2.084	1.615	2.400	3.138	1.866	2.320
Std. Error of Mean	0.5300	0.5706	0.6321	0.5054	0.5106	0.7237	0.7397	0.5176	0.6697
Mixed-effects analysis (within age-group)									
Table Analyzed	Ciliary Beating frequency								
Mixed-effects model (REML)	Matching:								

	Across row								
Assume sphericity?	No								
Alpha	0.05								
Fixed effect (type III)	P value	P value summary	Statistically significant (P < 0.05)?	F (DFn. DFd)	Geisser-Greenhouse's epsilon				
Treatment (between columns)	0.8039	ns	No	F (4.844 . 60.55) = 0.4531	0.6055				
Random effects	SD	Variance							
Individual (between rows)	0.7049	0.4969							
Residual	2.214	4.904							
Was the matching effective?									
Chi-square. df	2.165. 1								
P value	0.1412								
P value summary	ns								
Is there significant matching (P < 0.05)?	No								
Multiple comparisons (within age-group)									
Šídák's multiple comparisons test	Mean Diff.	95.00% CI of diff.	Below threshold?	Summary	Adjusted P Value				
48 -Aboral vs. 48 - Side	-0.1375	-2.870 to 2.595	No	ns	>0.9999	A-B			
48- Aboral vs. 48 - Oral	0.04545	-3.023 to 3.114	No	ns	>0.9999	A-C			
48 -Side vs. 48 - Oral	0.2000	-3.177 to 3.577	No	ns	>0.9999	B-C			
72 -Aboral vs. 72 - Side	0.2900	-2.835 to 3.415	No	ns	>0.9999	D-E			
72 -Aboral vs. 72 - Oral	0.1636	-3.173 to 3.501	No	ns	>0.9999	D-F			
72 - Side vs. 72 - Oral	0.1200	-1.996 to 2.236	No	ns	>0.9999	E-F			
96 -Aboral vs. 96 - Side	0.8308	-2.685 to 4.346	No	ns	0.9948	G-H			

96 -Aboral vs. 96 - Oral	1.133	-1.878 to 4.145	No	ns	0.8979	G-I			
96 - Side vs. 96 - Oral	0.5000	-2.615 to 3.615	No	ns	0.9997	H-I			

Table S19: Descriptive statistics. Brown-Forsythe ANOVA and Dunnett's T3 multiple comparisons test related to Figure S2B.

Descriptive statistics: Body length (µm)					
	48 hpf	72 hpf	96 hpf		
Number of values	40	39	38		
Mean	225.0	236.7	245.3		
Std. Deviation	22.10	34.14	34.09		
Std. Error of Mean	3.494	5.467	5.530		
Brown-Forsythe ANOVA test					
F* (DFn. DFd)	4.290 (2.000. 100.8)				
P value	0.0163				
P value summary	*				
Significant diff. among means (P < 0.05)?	Yes				
Welch's ANOVA test					
W (DFn. DFd)	5.190 (2.000. 71.51)				
P value	0.0079				
P value summary	**				
Significant diff. among means (P < 0.05)?	Yes				
Multiple comparisons					
Dunnett's T3 multiple comparisons test	Mean Diff.	95.00% CI of diff.	Below threshold?	Summary	Adjusted P Value
48 hpf vs. 72 hpf	-11.63	-27.51 to 4.257	No	ns	0.2137
48 hpf vs. 96 hpf	-20.26	-36.29 to -4.230	Yes	**	0.0087
72 hpf vs. 96 hpf	-8.631	-27.61 to 10.34	No	ns	0.6086

Table S20: Descriptive statistics. Brown-Forsythe ANOVA and Dunnett's T3 multiple comparisons test related to Figure 3B.

Descriptive statistics: Body axis ratio					
Age	48 hpf	72 hpf	96 hpf	120 hpf	
Number of values	37	33	31	31	
Mean	1.172	1.333	1.379	2.082	
Std. Deviation	0.1499	0.1762	0.1657	0.8098	
Std. Error of Mean	0.02464	0.03067	0.02976	0.1454	
Brown-Forsythe ANOVA test					
F* (DFn. DFd)	28.06 (3.000. 37.49)				
P value	<0.0001				
P value summary	***				

Significant diff. among means (P < 0.05)?	Yes				
Welch's ANOVA test					
W (DFn. DFd)	20.71 (3.000. 66 .65)				
P value	<0.0001				
P value summary	***				
Significant diff. among means (P < 0.05)?	Yes				
Multiple comparisons					
Dunnett's T3 multiple comparisons test	Mean Diff.	95.00% CI of diff.	Below threshold?	Summary	Adjusted P Value
48 vs. 72	-0.1611	-0.2678 to -0.05437	Yes	***	0.0007
48 vs. 96	-0.2066	-0.3115 to -0.1017	Yes	***	<0.0001
48 vs. 120	-0.9101	-1.322 to -0.4980	Yes	***	<0.0001
72 vs. 96	-0.04550	-0.1615 to 0.07046	No	ns	0.8661
72 vs. 120	-0.7490	-1.163 to -0.3345	Yes	***	<0.0001
96 vs. 120	-0.7035	-1.117 to -0.2895	Yes	***	0.0002
Compact letter display					
48hpf	A				
72hpf	B				
96hpf	B				
120hpf	C				

Table S21: Descriptive statistics. Kruskal Wallis test and Dunn's multiple comparisons test related to Figure 3G.

Relative frequency (%) Straight:				
Descriptive statistics:				
	48hpf	72hpf	96hpf	120hpf
Number of values	70	80	84	80
Mean	15.50	27.33	40.06	29.51
Std. Deviation	31.93	37.38	40.04	39.13
Std. Error of Mean	3.816	4.180	4.369	4.375
Kruskal Wallis results				
P value	<0.0001			
Exact or approximate P value?	Approximate			
P value summary	***			
Do the medians vary signif. (P < 0.05)?	Yes			
Number of groups	4			
Kruskal-Wallis statistic	22.14			
Multiple comparisons				
Dunn's multiple comparisons test	Mean rank diff.	Significant?	Summary	Adjusted P Value
72hpf vs. 48hpf	35.01	No	ns	0.0664
96hpf vs. 48hpf	64.08	Yes	***	<0.0001
120hpf vs. 48hpf	36.26	No	ns	0.0509
96hpf vs. 72hpf	29.07	No	ns	0.1623
120hpf vs. 72hpf	1.256	No	ns	>0.9999
120hpf vs. 96hpf	-27.82	No	ns	0.2065
Compact letter display				
48hpf	A			
72hpf	AB			
96hpf	B			
120hpf	AB			
Relative frequency (%) Turn:				
Descriptive statistics:				
Age	48hpf	72hpf	96hpf	120hpf
Number of values	70	80	84	80
Mean	9.227	19.44	19.81	17.50
Std. Deviation	16.27	21.00	21.37	25.16
Std. Error of Mean	1.944	2.348	2.332	2.813
Kruskal Wallis results:				
P value	0.0001			
Exact or approximate P value?	Approximate			
P value summary	***			
Do the medians vary signif. (P < 0.05)?	Yes			
Number of groups	4			
Kruskal-Wallis statistic	20.64			

Multiple comparisons				
Dunn's multiple comparisons test	Mean rank diff.	Significant?	Summary	Adjusted P Value
72hpf vs. 48hpf	53.83	Yes	**	0.0011
96hpf vs. 48hpf	57.67	Yes	***	0.0003
120hpf vs. 48hpf	29.46	No	ns	0.2411
96hpf vs. 72hpf	3.843	No	ns	>0.9999
120hpf vs. 72hpf	-24.37	No	ns	0.4738
120hpf vs. 96hpf	-28.21	No	ns	0.2373
Compact letter display				
48hpf	A			
72hpf	B			
96hpf	B			
120hpf	AB			
Relative frequency (%) Swirl:				
Descriptive statistics:				
Age	48hpf	72hpf	96hpf	120hpf
Number of values	70	80	84	80
Mean	12.72	23.45	21.53	10.39
Std. Deviation	27.77	32.57	33.46	24.86
Std. Error of Mean	3.319	3.642	3.651	2.779
Kruskal Wallis results				
P value	0.0028			
Exact or approximate P value?	Approximate			
P value summary	**			
Do the medians vary signif. (P < 0.05)?	Yes			
Number of groups	4			
Kruskal-Wallis statistic	14.08			
Multiple comparisons				
Dunn's multiple comparisons test	Mean rank diff.	Significant?	Summary	Adjusted P Value
72hpf vs. 48hpf	29.23	No	ns	0.0801
96hpf vs. 48hpf	24.07	No	ns	0.2361
120hpf vs. 48hpf	-6.477	No	ns	>0.9999
96hpf vs. 72hpf	-5.160	No	ns	>0.9999
120hpf vs. 72hpf	-35.71	Yes	*	0.0105
120hpf vs. 96hpf	-30.55	Yes	*	0.0405
Compact letter display				
48hpf	AB			
72hpf	A			
96hpf	A			
120hpf	B			

Relative frequency (%) Pause:				
Descriptive Statistics				
	48hpf	72hpf	96hpf	120hpf
Number of values	70	80	84	80
Mean	62.54	29.78	18.59	42.60
Std. Deviation	47.34	43.16	35.98	47.77
Std. Error of Mean	5.658	4.826	3.926	5.341
Kruskal Wallis results				
P value	<0.0001			
Exact or approximate P value?	Approximate			
P value summary	***			
Do the medians vary signif. (P < 0.05)?	Yes			
Number of groups	4			
Kruskal-Wallis statistic	30.50			
Multiple comparisons:				
Dunn's multiple comparisons test	Mean rank diff.	Significant?	Summary	Adjusted P Value
72hpf vs. 48hpf	-51.54	Yes	***	0.0009
96hpf vs. 48hpf	-71.35	Yes	***	<0.0001
120hpf vs. 48hpf	-32.48	No	ns	0.1000
96hpf vs. 72hpf	-19.81	No	ns	0.7563
120hpf vs. 72hpf	19.06	No	ns	0.8760
120hpf vs. 96hpf	38.87	Yes	*	0.0161
Compact letter display				
48hpf	A			
72hpf	BC			
96hpf	C			
120hpf	AB			

Table S22: Spearman's Rank correlations related to Figure 3H.

Spearman R correlations: AR vs speed			
Age	State	Correlation coefficient R^2	p-value
"48hpf"	"Straight"	-0.281625075578344	8.25394271109857e-22
"48hpf"	"Turn"	0.521623978070662	8.61889617192878e-37
"48hpf"	"Swirl"	0.716538956841716	1.68892753383344e-165
"48hpf"	"Pause"	-0.0327360860232468	0.697916160130748
"72hpf"	"Straight"	0.252142391039874	2.67020884484153e-34
"72hpf"	"Turn"	0.489628269515687	4.85582676432303e-60
"72hpf"	"Swirl"	0.468793917994718	2.44282166667514e-99
"72hpf"	"Pause"	0.0339179732723225	0.721356246776579
"96hpf"	"Straight"	0.593274497851232	7.75693203035617e-310
"96hpf"	"Turn"	0.348489428921122	7.94468833754512e-32
"96hpf"	"Swirl"	0.401931949653022	2.33790855198034e-84
"96hpf"	"Pause"	0.228549462542341	0.0215152065492429

"120hpf"	"Straight"	0.380330036346669	3.24170052303052e-54
"120hpf"	"Turn"	-0.178502045922531	1.6139467592037e-06
"120hpf"	"Swirl"	0.119049789946587	0.00100803316535083
"120hpf"	"Pause"	0.525319353246788	5.20663866569449e-16
Linear regression:	Model:	lm (speed ~ AR * Age)	
State	Age	Slope	
Straight	48hpf	-190.2	
Straight	72hpf	508.1	
Straight	96hpf	885	
Straight	120hpf	156.8	
Turn	48hpf	456.6	
Turn	72hpf	706.5	
Turn	96hpf	459.4	
Turn	120hpf	-166.6	
Swirl	48hpf	868.6	
Swirl	72hpf	1241	
Swirl	96hpf	707.5	
Swirl	120hpf	144.6	
Pause	48hpf	-8	
Pause	72hpf	-10.5	
Pause	96hpf	106.1	
Pause	120hpf	6.1	

Table S23: Spearman's Rank correlations related to Figure S3A.

	48hpf mean Area vs. 48hpf mean AR	72hpf mean Area vs. 72hpf mean AR	96hpf mean Area vs. 96hpf mean AR	120hpf mean Area vs. 120hpf mean AR
Spearman r				
r	0.2329	0.08908	0.1291	0.7871
95% confidence interval	-0.005698 to 0.4464	-0.1397 to 0.3089	-0.09410 to 0.3400	0.6826 to 0.8601
P value				
P (two-tailed)	0.0490	0.4320	0.2418	<0.0001
P value summary	*	ns	ns	***
Exact or approximate P value?	Approximate	Approximate	Approximate	Approximate
Significant? (alpha = 0.05)	Yes	No	No	Yes
Number of XY Pairs	72	80	84	80

Table S24: Spearman’s Rank correlations related to Figure S3B.

	48hpf mean Area vs. 48hpf Mean speed	72hpf mean Area vs. 72hpf Mean speed	96hpf mean Area vs. 96hpf Mean speed	120hpf mean Area vs. 120hpf Mean speed
Spearman r				
r	0.1488	0.02375	-0.1061	-0.3973
95% confidence interval	-0.09276 to 0.3738	-0.2033 to 0.2484	-0.3191 to 0.1172	-0.5719 to -0.1882
P value				
P (two-tailed)	0.2123	0.8344	0.3368	0.0003
P value summary	ns	ns	ns	***
Exact or approximate P value?	Approximate	Approximate	Approximate	Approximate
Significant? (alpha = 0.05)	No	No	No	Yes
Number of XY Pairs	72	80	84	80

Table S25: List of gene markers related to Figure 4C.

Gene markers	
NV2.geneid	gene.name
NV2.19947	Nav2.4
NV2.20610	Nav2.5
NV2.23334	Nav2.1
NV2.252	elavl3
NV2.15016	GP2_15016
NV2.14524	GP2_14524
NV2.8021	Prd146

Table S26: Descriptive statistics. Wilcoxon matched pairs signed rank test related to Figure 4H.

Descriptive statistics: CBF (Hz) of pipette-attached larvae				
	CBF 1 control	CBF 1 mgcl2	CBF 2 control	CBF 2 mgcl2
Number of values	4	5	4	5
Mean	14.05	13.76	13.50	13.42
Std. Deviation	0.8699	0.7765	0.6481	0.9445
Std. Error of Mean	0.4349	0.3473	0.3240	0.4224
Wilcoxon test:				
Group B	CBF 2 control	CBF 2 mgcl2		
vs.	vs.	vs.		
Group A	CBF 1 control	CBF 1 mgcl2		
Wicoxon matched-pairs signed rank test				
P value	0.2500	0.8750		
Exact or approximate P value?	Exact	Exact		
P value summary	ns	ns		
Signficantly different (P < 0.05)?	No	No		
One- or two-tailed P value?	Two-tailed	Two-tailed		
Sum of positive. negative ranks	1.000 . -9.000	4.000 . -6.000		
Sum of signed ranks (W)	-8.000	-2.000		
Number of pairs	4	5		

Number of ties (ignored)	0	1		
Mean of differences				
Median	0.7500	0.000		
How effective was the pairing				
rs (Spearman)	0.4000	0.3078		
P value (one tailed)	0.3750	0.3000		
P value summary	ns	ns		
Was the pairing significantly effective?	No	No		

Table S27: Quantification of moving vs stationary larvae and Two-proportion Z-test related to Figure 4I.

Quantification of moving vs stationary larvae					
Treatment	# stationary	# moving	Total #	% stationary	% moving
Control	17	67	84	20	80
MgCl2	30	58	88	34	66
Two tailed two-proprotion Z-test:					
Z-statistic	2.06339				
p-value	p=0.03908				

Table S28: Descriptive statistics and Kolmogorov-Smirnov test related to Figure 4J.

Moving larvae max speed (µm/sec)		
Descriptive statistics		
Treatment	Ctrl	MgCl
Number of values	67	58
Mean	693.1	854.5
Std. Deviation	288.1	336.1
Std. Error of Mean	35.19	44.13
Kolmogorov Smirnov test		
Comparison	D-statistic	p.value
Moving larvae Ctrl vs MgCl	0.32861	0.001599

Table S29: Descriptive statistics and Kolmogorov-Smirnov test related to Figure 4K.

All larvae mean axis ratio		
Descriptive statistics		
Treatment	Ctrl AR	MgCl AR
Number of values	75	84
Mean	1.092	1.219
Std. Deviation	0.08148	0.1720
Std. Error of Mean	0.009408	0.01877
Kolmogorov Smirnov test		
Comparison	D-statistic	p.value
All larvae Ctrl vs MgCl	0.40881	2.2e-1

Table S30: Quantification of Elav::mOrange positive neurons related to Figure S4A.

Neuron Quantification					
Age	#slices in stack	Slice depth (µm)	Depth (µm)	Average neuronal count	Average neurons per µm depth
48hpf	60	1.2	72	17	0.236111111
48hpf	70	1.2	84	26	0.30952381
48hpf	85	1.2	102	43	0.421568627
72hpf	39	1.2	46.8	140	2.991452991
72hpf	39	1.2	46.8	153.5	3.27991453
72hpf	33	1.2	39.6	73	1.843434343
72hpf	32	1.2	38.4	99	2.578125
72hpf	30	1.2	36	118.5	3.291666667
96hpf	60	1.2	72	99.5	1.381944444
96hpf	69	1.2	82.8	166	2.004830918
96hpf	40	1.2	48	136	2.833333333
96hpf	41	1.2	49.2	65.5	1.331300813
96hpf	30	1.2	36	125.5	3.486111111
120hpf	37	1.2	44.4	121.5	2.736486486
120hpf	84	1.2	100.8	188	1.865079365
120hpf	89	1.2	106.8	304.5	2.851123596
120hpf	46	1.2	55.2	394	7.137681159
120hpf	57	1.2	68.4	478.5	6.995614035
120hpf	49	1.2	58.8	422.5	7.18537415

Table S31: TRP channels candidates related to Figure S4B.

TRP channels candidates					
NV2.geneid	top.human	top.human.fam	top.human.subfam	gene.name	NVE.geneid
NV2.22582	TRPA1	TRP	TRPA	TRPA1-like-11	NVE25611
NV2.24495	TRPC5	TRP	TRPC	TRPC5-like-12	NVE17429. NVE17428. NVE17427
NV2.7365	PKD2L1	TRP	TRPP	PKD2-like-3	NVE4860
NV2.8602	TRPA1	TRP	TRPA	TRPA1-like-25	NVE1852
NV2.2930	PKD2	TRP	TRPP	PKD2-like-2	NVE5442
NV2.6626	TRPC5	TRP	TRPC	TRPC5-like-13	NVE17150. NVE17151. NVE17152
NV2.7252	TRPC4	TRP	TRPC	TRPC3-like-1	NVE21830. NVE24126
NV2.18039	TRPC5	TRP	TRPC	TRPC3-like-2	NVE16441
NV2.16727	TRPA1	TRP	TRPA	ANR28-like-4	NVE16222. NVE3353
NV2.15467	TRPA1	TRP	TRPA	ANK3-like-3	NVE25994. NVE25995
NV2.10225	TRPC6	TRP	TRPC	TRPC2-like-1	NVE2281
NV2.15790	TRPA1	TRP	TRPA	ASB8-like-1	NVE733
NV2.521	TRPV1	TRP	TRPV	TRPV5-like-1	NVE1085. NVE1087
NV2.519	PKD2L2	TRP	TRPP	TRPV5-like-2	NVE25977. NVE1088
NV2.12744	TRPM2	TRP	TRPM	TMP2L-like-2	NVE21472
NV2.23620	TRPM3	TRP	TRPM	TRPM3-like-2	NVE2243. NVE2244
NV2.8726	TRPM3	TRP	TRPM	TRPM3-like-1	NVE21538. NVE21537
NV2.8930	TRPV6	TRP	TRPV	TRPV6-like-6	NVE6505. NVE6506
NV2.1902	MCOLN3	TRP	TRPML	MCLN3-like-1	NVE20480
NV2.1904	MCOLN3	TRP	TRPML	MCLN3-like-2	NVE20482
NV2.7219	PKD2L1	TRP	TRPP	PKD2-like-19	NVE24095
NV2.16659	PKD2L1	TRP	TRPP	PK1L2-like-43	NVE15023
NV2.7555	PKD2	TRP	TRPP	PKD2-like-17	NVE22120. NVE2772
NV2.20195	PKD2L2	TRP	TRPP	PKD2-like-10	NVE23138
NV2.18254	PKD2L1	TRP	TRPP	PK1L2-like-15	NVE9510
NV2.21369	PKD2	TRP	TRPP	PKD2-like-23	NVE14122
NV2.20496	PKD2L2	TRP	TRPP	PK1L2-like-16	NVE22456
NV2.18451	TRPA1	TRP	TRPA	TRPA1-like-41	NVE2073
NV2.6894	PKD2	TRP	TRPP	PK1L2-like-30	NVE399. NVE20238
NV2.18932	PKD2	TRP	TRPP	PK1L2-like-3	NVE21043
NV2.8942	TRPA1	TRP	TRPA	TRPA1-like-14	NVE6494. NVE13672
NV2.8357	TRPA1	TRP	TRPA	TRPA1-like-6	NVE7487
NV2.18388	TRPV6	TRP	TRPV	TRPV6-like-1	NVE4515
NV2.2120	PKD2	TRP	TRPP	PKD2-like-31	NVE15112. NVE11101. NVE6082
NV2.8911	TRPA1	TRP	TRPA	TRPA1-like-24	NVE1715. NVE1716
NV2.8294	TRPA1	TRP	TRPA	TRPA1-like-2.1	NVE23442. NVE23441. NVE23447. NVE23443
NV2.659	TRPA1	TRP	TRPA	TRPA1-like-45	NVE6201. NVE25743
NV2.103	TRPA1	TRP	TRPA	TRPA1-like-31	NVE16115. NVE16114
NV2.229	TRPA1	TRP	TRPA	TRPA1-like-39	NVE2486. NVE5579. NVE2483. NVE2484
NV2.13263	TRPA1	TRP	TRPA	TRPA1-like-40	NVE12216
NV2.642	TRPA1	TRP	TRPA	TRPA1-like-3	NVE6214
NV2.9107	TRPA1	TRP	TRPA	TRPA1-like-28	NVE1652. NVE1653
NV2.8359	TRPA1	TRP	TRPA	TRPA1-like-19	NVE16735. NVE7488
NV2.7363	TRPA1	TRP	TRPA	TRPA1-like-1	NVE4862. NVE20293

NV2.5171	TRPA1	TRP	TRPA	TRPA1-like-22	NVE3824. NVE3823
NV2.3562	TRPA1	TRP	TRPA	TRPA1-like-15	NVE9237
NV2.7513	TRPA1	TRP	TRPA	TRPA1-like-38	NVE25441
NV2.19377	TRPA1	TRP	TRPA	TRPA1-like-23	NVE1048. NVE1050
NV2.24937	TRPC4	TRP	TRPC	TRPC5-like-1	NVE18773. NVE18772. NVE18774
NV2.6623	TRPC5	TRP	TRPC	TRPC5-like-18	NVE17148. NVE17149. NVE5989. NVE5988
NV2.10352	TRPM7	TRP	TRPM	SPRY3-like-1	NVE24284

Table S32: Descriptive statistics and Kolmogorov-Smirnov test related to Figure S4D.

Moving larvae mean speed (μm/sec)		
Descriptive statistics		
Treatment	Ctrl	MgCl
Number of values	67	58
Mean	454.1	599.7
Std. Deviation	248.4	280.7
Std. Error of Mean	30.35	36.85
Kolmogorov Smirnov test		
Comparison	D-statistic	p.value
Moving larvae Ctrl vs MgCl	0.32861	0.001599

Table S33: List of mean speed quartiles related to Figure S4E.

Quartiles: Mean speed (μm/sec)			
	Control	MgCl2	Both
	(n=84)	(n=88)	(n=172)
Quartile			
Q1	[0-219) μm/sec	[0-66.4) μm/sec	[0-125.5) μm/sec
Q2	[219-324) μm/sec	[66.4-389) μm/sec	[125.5-359.1) μm/sec
Q3	[324-516) μm/sec	[389-718) μm/sec	[359.1-601.9) μm/sec
Q4	[516-1427] μm/sec	[718-1136.5] μm/sec	[601.9-1427] μm/sec

Table S34: Spearman's Rank correlations related to Figure S4F

Spearman correlation: Mean Axis ratio vs Mean speed		
Treatment	Correlation coefficient R^2	n
Ctrl	0.282	58
MgCl	0.255	67

Table S35: Spearman's Rank correlations and Linear regression analysis related to Figure S4G.

Spearman R correlation:	Axis ratio vs. Speed			
Treatment	State	R^2 spearman correlation	P value	
"Ctrl"	"Straight"	0.393788239826912	1.87031846493278e-107	
"Ctrl"	"Turn"	0.294038837030199	8.11709183041444e-18	
"Ctrl"	"Swirl"	-0.0381694492367463	0.0909007681942098	
"Ctrl"	"Pause"	0.238627713718055	0.0536619444254147	
"MgCl"	"Straight"	0.20500895247124	1.18234028935805e-41	
"MgCl"	"Turn"	0.190913158795879	7.65942413724253e-13	
"MgCl"	"Swirl"	0.228681482641556	6.74172534470556e-20	
"MgCl"	"Pause"	-0.0514125216386432	0.615132690983376	
Linear regression:				
Model:	*lm (speed ~ AR * Treatment)*			
State	Treatment	Slope	Slope difference (slope_MgCl − slope_Ctrl)	p-value from t-test
Straight	Ctrl	1144.96		
Straight	MgCl	247.07	-897.88	9.00E-85
Turn	Ctrl	1312.32		
Turn	MgCl	318.66	-993.66	3.10E-23
Swirl	Ctrl	498.37		
Swirl	MgCl	348.74	-149.63	0.15
Pause	Ctrl	45.53		
Pause	MgCl	-17.52	-63.04	0.46
